# Clusters versus Affinity-Based Approaches in *F. tularensis* Whole Genome Search of CTL Epitopes

**DOI:** 10.1371/journal.pone.0036440

**Published:** 2012-05-01

**Authors:** Anat Zvi, Shahar Rotem, Ofer Cohen, Avigdor Shafferman

**Affiliations:** Department of Biochemistry and Molecular Genetics, Israel Institute for Biological Research, Ness Ziona, Israel; Technical University of Denmark, Denmark

## Abstract

Deciphering the cellular immunome of a bacterial pathogen is challenging due to the enormous number of putative peptidic determinants. State-of-the-art prediction methods developed in recent years enable to significantly reduce the number of peptides to be screened, yet the number of remaining candidates for experimental evaluation is still in the range of ten-thousands, even for a limited coverage of MHC alleles. We have recently established a resource-efficient approach for down selection of candidates and enrichment of true positives, based on selection of predicted MHC binders located in high density “hotspots" of putative epitopes. This cluster-based approach was applied to an unbiased, whole genome search of *Francisella tularensis* CTL epitopes and was shown to yield a 17–25 fold higher level of responders as compared to randomly selected predicted epitopes tested in Kb/Db C57BL/6 mice. In the present study, we further evaluate the cluster-based approach (down to a lower density range) and compare this approach to the classical affinity-based approach by testing putative CTL epitopes with predicted IC_50_ values of <10 nM. We demonstrate that while the percent of responders achieved by both approaches is similar, the profile of responders is different, and the predicted binding affinity of most responders in the cluster-based approach is relatively low (geometric mean of 170 nM), rendering the two approaches complimentary. The cluster-based approach is further validated in BALB/c *F. tularensis* immunized mice belonging to another allelic restriction (Kd/Dd) group. To date, the cluster-based approach yielded over 200 novel *F. tularensis* peptides eliciting a cellular response, all were verified as MHC class I binders, thereby substantially increasing the *F. tularensis* dataset of known CTL epitopes. The generality and power of the high density cluster-based approach suggest that it can be a valuable tool for identification of novel CTLs in proteomes of other bacterial pathogens.

## Introduction

*F. tularensis*, the causative infective agent of the acute tularemia disease, is a facultative intracellular pathogen. The bacterium has a broad range of host specificities and its clinical manifestation and disease severity depend on the route of infection and on the infecting strain. Inhalation of as few as 15 CFU of the more virulent strains (*F. tularensis* tularensis type A) is sufficient to infect humans, and without treatment the mortality rate for respiratory disease is 30–60% [1], [2], [3]. This, together with interest in *F. tularensis* as a biological warfare agent led to its classification in category A of the CDC list of bioterrorism agents. To date, there is no available licensed vaccine against *F. tularensis*. The restricted efficacy of the sole prophylactic vaccine available, the attenuated live vaccine strain (LVS), motivated extensive research efforts attempted at identification of alternative vaccine formulations as a countermeasure, including exploration of different live and killed attenuated strains, and identification of components of subunit vaccines [4], [5], [6], [7], [8], [9], [10]. Taken together with the well documented evidence on contribution of the cellular response to protection against the intracellular *F. tularensis* [11], identification of determinants which could elicit an effective cellular-mediated immune response is crucial. Nevertheless, the knowledge on *F. tularensis* epitopes, and more specifically on cytotoxic T-cell (CTL) epitopes is extremely limited (www.immuneepitope.org). Recent studies targeted at large-scale identification of CTL epitopes in *F. tularensis* were restricted to limited pre-defined subsets of source proteins, preselected on the basis of particular considerations, e.g. secreted proteins [12], [13].

**Figure 1:**
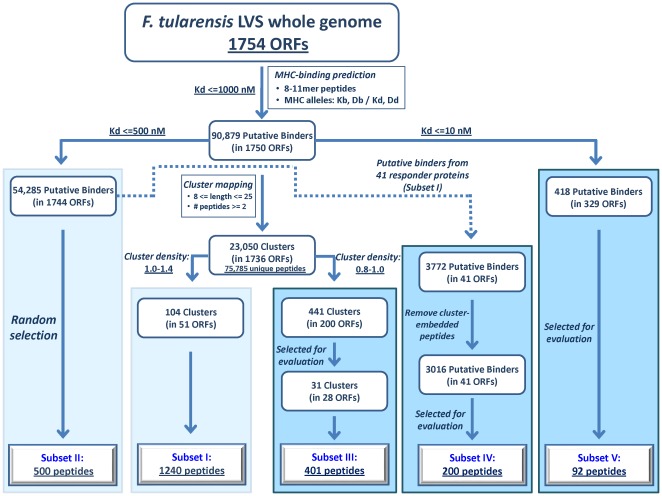
Flowchart of down-selection of putative MHC binders for experimental evaluation. The whole-genome immunoinformatic analysis conducted on the 1754 *F. tularensis* ORF products and the downstream selection of the various subsets of peptides is provided. Boxes shaded in light blue: Subsets I and II were described in a previous study [14] and are added for the sake of completeness. Boxes shaded in cyan: Subsets III–V of peptides selected and evaluated in this study. In all down selection steps referred to as : “Selected for evaluation" (in Subset III, IV and V), clusters or peptides are selected at random as an operational filter for reduction of number of peptides to be experimentally tested, unlike Subset II, were selection of peptides was at random with respect to clusters. Subset III contains 401 peptides selected from clusters having density of 0.8 up to 1.0 (1.0 not included); Subset IV is composed of 200 peptides selected from the overall 3016 predicted MHC binders (IC_50_< = 500 nM) located outside the high-density cluster regions in the 41 source proteins of Subset I (Figure 2); Subset V includes 92 predicted epitopes having an IC_50_ of 10 nM and lower, representing putative high-affinity MHC binders.

Recently, we developed and established an approach for a whole genome immune-analysis of a bacterial genome, aiming at the unbiased selection of putative CTL epitopes [14]. The approach is based on mapping of immunological “hotspots" (regions of 8–25 amino acids harboring consecutive predicted MHC binders, denoted as clusters), and selection of highly-dense epitope clusters for further experimental evaluation (the density of a cluster being defined as the number of peptides per cluster length, and the densities of the clusters selected range from 1.0–1.4). A total of 1240 peptides were tested by an EliSpot assay for their potential to elicit an immune response as reflected by their ability to stimulate lymphocytes and induce IFNγ production (such peptides are referred in the text as responders). This screen resulted in identification of 127 responders, which constitute 10.2% of the tested peptides. The cluster-based method was demonstrated to be a strategy for enrichment (17–25 fold) of true positive responders in comparison to a randomly selected set of predicted peptides which are unrelated to clusters. The method was first applied for clusters harboring a very high density of putative MHC binders, which in turn were found to reside in a limited set of proteins (51 proteins, see Figure 1, Subset I). Along with the high success rate obtained by the cluster-based method, some important issues remained to be further clarified in order to determine both the generality of the approach developed as well as to compare its performance with respect to an alternative approach of high affinity-based selection. Accordingly, the following topics were evaluated experimentally:

1. Investigating the effect of extension of both the protein number covered by the selected putative epitopes, and the range of densities tested (0.8–1.0).
2. Examining whether the high success rate obtained by the cluster-based approach is indeed related to the epitope clustering and not to some unique characteristics of the parental proteins (e.g., membranal).
3. Assessing the success rate of the cluster-based approach in comparison to the conventional, well established strategy based solely on the predicted binding affinity of the peptide to the MHC molecule (10 nM and lower).
4. Validating the cluster-based approach in another animal model with a different allelic restriction - BALB/c, in addition to the original C57BL/6 model.

In order to address all four issues raised above, nearly 700 additional predicted MHC binders were synthesized and tested for their ability to elicit a cellular response. The results obtained in the present study strongly support and reinforce the strength of the approach of selecting putative CTL epitopes from epitope-rich clusters as both a reductive/filtering and enrichment method of CTL positive responders. This study also provides insight into the potential of the cluster-based approach and its added value in relation to the conventional high affinity-based approach.

## Results

The general strategy employed for selection of putative peptides for experimental evaluation is summarized in the flowchart presented in Figure 1. Our previous study described in detail the screen conducted on 1740 putative CTL epitopes, preselected from a total of 90,879 predicted MHC binders [14]. The 1740 putative epitopes constitute two subsets of peptides: (I) *Subset I* - was derived from regions rich in overlapping peptides predicted to be MHC binders with binding affinity threshold of 1000 nM. These regions were denoted as “hotspots" or clusters of epitopes, operationally defined as polypeptides of 8–25 amino acids, harboring at least 2 consecutive predicted epitopes. Clusters were further ranked according to their calculated density (i.e. number of putative epitopes per cluster length), and top-ranking clusters having densities 1.0–1.4 were further considered, encompassing a total of 1240 peptides; (II) *Subset II* - included 500 predicted MHC binders which were randomly selected, regardless of their location in clusters, and evaluated as a validation control set to the cluster-based selection approach (Figure 1). In the current study, we tested the potential of additional categories of preselected peptides, which were divided into three peptide subsets (Subset III, Subset IV and Subset V – Figure 1). These subsets were used to address the issues raised in the Introduction, regarding the generality of the cluster-based approach and its potential strength and/or weakness as compared to the high affinity-based selection approach (as detailed below). The new Subsets III, IV and V were evaluated experimentally with regard to their potential to elicit a T-cell response. In all down selection steps referred to in Figure 1 as : “Selected for evaluation" (Subset III, IV and V), the clusters or peptides are selected at random as an operational filter for reduction of number of peptides to be experimentally tested, unlike Subset II, where selection of peptides was at random with respect to clusters. In addition to Subsets I–V which were all evaluated on C57BL/6 mice (Kb/Db alleles), we selected for analysis a set of peptides for evaluation in BALB/c mice which belong to a different allelic group (Kd/Dd). Experimental evaluation of all various subsets was carried out using splenocytes isolated from mice (C57BL/6 or BALB/c, according to the peptide subset) intranasally immunized with a sub-lethal dose of live LVS. These splenocytes were co-incubated with the individual peptides, and the extent of stimulation and subsequent IFNγ production was assessed by an EliSpot assay (Materials & Methods).

### Assessment of the cluster-based approach in a density range of 0.8–1.0 (Subset III)

**Figure 2:**
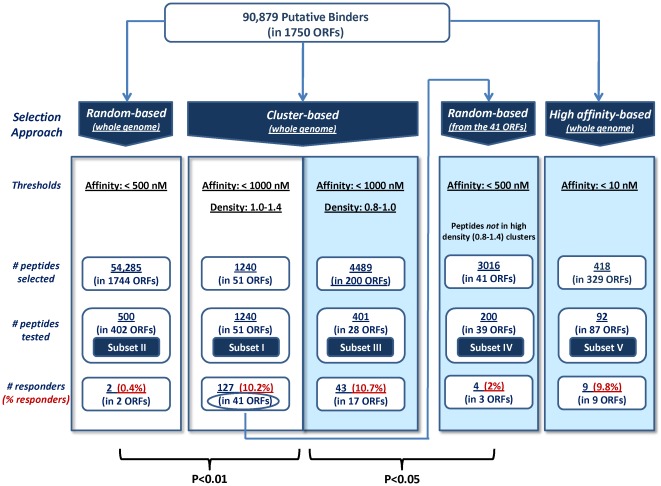
Array of peptides tested in the *F. tularensis* CTL screen in C57BL/6 LVS immunized mice. Summary of the total number of peptides selected, evaluated and responding in the various subsets (see Figure 1). Data on Subset I and II is from [14]. Peptides which were shown to stimulate lymphocytes and induce IFNγ production are referred to as “responders". P-values are provided for evaluation of significance of difference between number of responders in the cluster-based Subsets I and III versus Subset II, and versus Subset IV.

**Figure 3:**
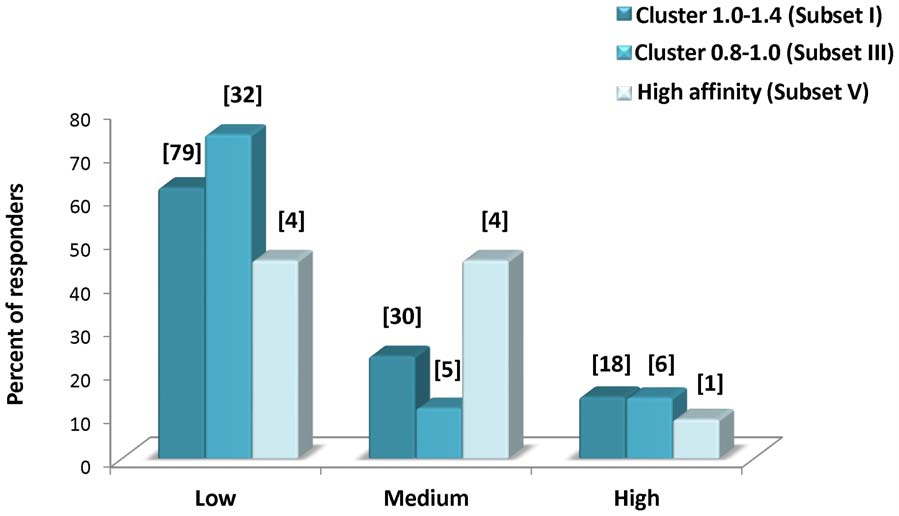
Magnitude of T-cell response among identified CTL epitopes (Subsets I, III, V). Responders identified from clusters with densities of 1.0–1.4 (dark cyan) (determined from [14]), from clusters with densities of 0.8–1.0 (light cyan) and from the high affinity-based approach (light blue) are classified according to magnitude of T-cell response, as deduced from number of spots in the EliSpot assay. The bars represent the percent of responders per subset in each of the classes, while the actual number of responders is given in brackets on the top of the corresponding bar. Classification (expressed in number of spots/million cells) is: Low: 5–20; Medium: 20–32; High> = 33).

Out of a total of 23,050 mapped clusters of putative epitopes, only 104 clusters exhibited a density of 1.0–1.4 (e.g. a given cluster of 25 amino acids contains 25–35 predicted epitopes). We now expand the range of cluster densities to less than 1.0 down to 0.8 (this is referred in the text as Subset III, and denoted as density range 0.8–1.0), allowing to cover a larger number of clusters and source proteins. In this range of densities, we identified 441 clusters which contain 4489 peptides residing in 200 proteins (Figure 1). It should be emphasized that such cluster densities (0.8–1.0) still contain a rather large number of peptides per cluster (i.e. 20–22 peptides of 8–11 amino acids per fragment of 25 amino acids). Out of the 441 clusters, we selected for experimental evaluation 31 clusters encompassing an overall of 401 peptides contained within 28 proteins (Figure 1). The 401 peptides were analyzed for their ability to elicit a specific T-cell response (IFNγ production). This screen resulted in an overall of 43 responders (distributed in 17 proteins), which constitute 10.7% of all peptides tested in this subset (Figure 2 and Table S1). These findings further corroborate the strength of the cluster-based approach as an approach for selection and enrichment of true positives, and validate the procedure, which was first exemplified for the highest density clusters (1.0–1.4, Subset I, [14]), to the somewhat lower densities (Subset III). The distribution of the epitopes according to the extent of response (as measured by the number of spots counted in a quantitative EliSpot assay, see Materials & Methods) was generally found to be similar for the responders in Subset III as for the responders in Subset I (Figure 3), where ∼20% of the responders induce production of IFNγ at very high level (> = 33 spots in the EliSpot assay).

**Figure 4:**
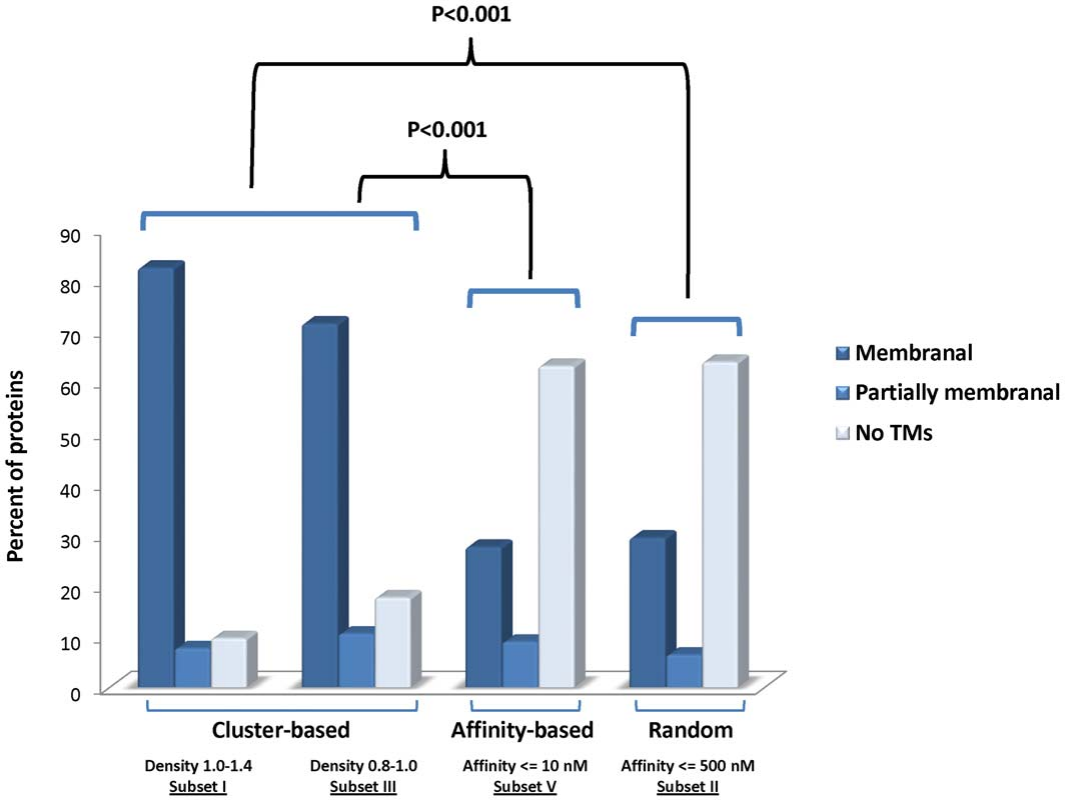
Distribution of parental proteins containing responders according to their membrane topology. Parental proteins containing responder peptides identified by the cluster-based, high affinity-based and random-based approaches are classified as follows: Membrane-spanning proteins (with 10 or more predicted transmembranal domains (TM) and/or TMs spanning at least a third of the protein) in dark blue; partially membranal proteins (less than 10 predicted TMs) in blue; proteins with no predicted transmembranal domains (except signal peptide) in light blue. . P-values are provided for evaluation of significance of difference between membranal proteins in the cluster-based Subsets I and III versus Subset V and versus Subset II.

We previously reported on a clear bias toward membranal proteins detected for the 51 parental proteins of Subset I (cluster densities 1.0–1.4) [14]. It was therefore interesting to determine if such a bias exists for the 28 proteins antigens of Subset III, which contain the clusters at lower densities of 0.8–1.0. Accordingly, these 28 proteins were investigated for their structured topology by prediction of transmembranal helices (see Materials and Methods and Figure 4). The analysis reveals that about 72% of the above mentioned 28 proteins are assigned as membranal proteins, similar to the results obtained for the proteins in Subset I. This percentage of membranal proteins is significantly higher (p<0.001) than that observed for the whole genome (∼30%) or for the group of proteins in which the randomly selected predicted epitopes reside (Subset II) (Figure 4).

### Evaluation of predicted MHC binders located outside of the high density clusters (1.0–1.4) that contain responders (Subset IV)

The fact that the high density clusters that contain responders reside in a relatively low number of proteins (41 ORF products), as well as the structural bias of these 41 proteins (membrane-spanning, see above) motivated us to determine whether the screen based on epitope “hotspots" mapping resulted in convergence to proteins which are immunogenic by nature. In such a case, putative MHC peptides derived from these proteins could have had a high probability of being immune responders, irrespective of their location in or outside of a cluster of epitopes. The total number of predicted MHC binders (with an IC_50_ value of 500 nM and lower) contained in the 41 proteins is 3722 (Figure 1). Of these 3772 peptides, 3016 peptides are located outside the high density (0.8–1.4) cluster regions (Figure 2). We randomly selected out of these putative 3016 peptides 200 peptides for experimental evaluation, and designated these as subset IV (Figures I and 2). Out of the 200 peptides tested, only 4 peptides (2%) were found to be responders (Figure 2 and Table S2). This percentage of response is by far lower than the extent of response observed for peptides in Subset I (10.2%) originating from high density cluster regions within the evaluated 41 proteins. These results clearly demonstrate that the major factor contributing to the enrichment of responders is the clustering of epitopes rather than the nature of the protein in which they are located. Nevertheless, one could argue that the rather higher percentage of responder peptides obtained in Subset IV (2%) in comparison to Subset II (0.4%) might reflect a certain contribution of the protein nature to the extent of response of its derived peptides (we note that the peptides of subset II and subset IV were both selected at random from a pool of putative MHC binders with predicted IC_50_ of 500 nM or lower). Yet, the small number of responders in these two groups (Subsets II and IV) does not allow for a statistically significant conclusion. On the other hand, the results from both of these subsets which include analysis of 700 putative MHC binders permit to estimate with some confidence that the fraction of responders among all predicted MHC binders in this bacterial genome is within a range of 0.4–2%.

### Conventional affinity-based screening strategy versus the cluster-based approach (Subset V)

To further evaluate the performance of the cluster-based approach as a filtering and enrichment strategy for identification of CTL epitopes, we compared this approach to the classical affinity-based approach. The classical approach relies solely on the high predicted binding affinity of the peptides, based on the documented correlation between MHC binding affinity and immunogenicity [15], [16], [17], [18]. Practically, a “strong" binder is commonly defined as a peptide having an IC_50_ value of 50 nM and lower. We chose to concentrate on a subset of peptides with a predicted binding affinity of 10 nM at most. In accordance with the experimental model (C57BL/6 mice), we focused on peptides predicted to bind Kb and/or Db MHC alleles. Accordingly, a total of 418 peptides predicted to have IC_50_ values of 10 nM or less were identified in the whole genome of *F. tularensis* and from these we chose at random 92 peptides for subsequent evaluation (Subset V, Figure 1). As could be expected, the 92 peptides were distributed in a relatively large number of proteins (87 proteins), and of these 92 peptides, 9 peptides (9.8%) were found to be responders (Figure 2 and Table S3). This percent of response is similar to that obtained for peptides originating from density-rich clusters (10.2–10.7%; subsets I and III). It was also interesting to determine the distribution of the responder peptides of Subset V with respect to the intensity of the response obtained, and to compare it to the distribution in the cluster-based Subsets I and III, (Figure 3). It appears that among the three Subsets I, III and V, the pattern of distribution is by and large similar and the proportion of peptides with high level of response is 10–20% and about 50–70% have a rather low intensity of response. Yet, it should be noted that the total number of responders in the high affinity-based subset (Subset V) is smaller by an order of magnitude than the number of responders in the cluster-based subset (Subset III).

### Validation of the cluster-based approach in the BALB/c model

The approach of cluster-based selection of putative MHC binders was established and proven on C57BL/6 mice immunized with the LVS *F. tularensis* strain ([14] and previous sections of this manuscript). In order to examine the generality of this approach, we repeated the evaluation of putative epitope located in highly dense regions of predicted binders in a different allelic group background. Out of the 545 clusters harboring a density of 0.8–1.4, 484 clusters possess peptides with BALB/c restriction, of which we selected 262 peptides for experimental evaluation on splenocytes isolated from LVS immunized BALB/c mice (Table S4). An additional pool of 108 peptides selected by the high affinity-based approach described above and having a predicted IC_50_ value lower than 10 nM, was tested in the BALB/c model (Table S4). As shown in Table 1, the percent of responders obtained is similar to values observed for the C57BL/6 model, namely 11.1% vs. 10.4% for the cluster-based selected peptides and 7.4% vs. 9.8% for the high affinity-based selected peptides.

**Table 1:** Comparison of cluster-based and high affinity-based screens conducted in C57BL/6 and BALB/c mouse models.

*Selection Approach*	Cluster-based		High affinity-based	
	(density 0.8–1.4)		(affinity< = 10 nM)	
*Mouse model*	**C57BL/6**	**BALB/c**	**C57BL/6**	**BALB/c**
*# Peptides evaluated*	1641	262	92	108
*# Responders*	170	29	9	8
*% Responders*	**10.40%**	**11.10%**	**9.8%**	**7.40%**

### Assessment of the T-cell population involved in the peptide mediated cellular response

**Figure 5:**
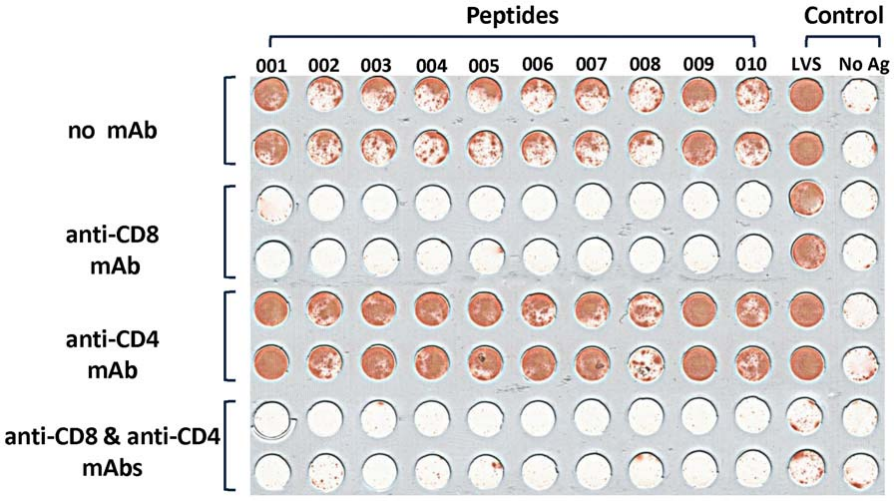
Effect of anti-CD8 and anti-CD4 antibodies on the T-cell response. A sample of over 20 EliSpot plates is presented, in which each of the first ten columns contains splenocytes incubated with an individual peptide and the last two columns are positive and negative controls, containing splenocytes incubated with formalin killed LVS (**LVS**) and splenocytes devoid of antigen (**No Ag**), respectively. Rows 1 and 2 (duplicates) contain splenocytes incubated with peptides denoted 001-010 (arbitrary numbering), or with LVS; the following pairs of rows (from top to bottom) are repeats of rows 1 and 2 supplemented with anti-CD8, anti-CD4 or a mixture of anti-CD4 and anti-CD8 antibodies. All 222 *F. tularensis* responder peptides exhibited the same behavior as depicted in the sample plate shown in this figure, with respect to the effect of anti-CD8 and anti-CD4 antibodies (see text and (Table S5)).

The cellular response elicited by the immunogenic peptides, predicted to be MHC class I binders, was determined by following the production of IFNγ. Yet, an ultimate and decisive verification of whether IFNγ secretion is exclusively mediated by cytotoxic T-cells and not by helper T-cells can be accomplished by addition of antibodies inhibiting the IFNγ secreted by either pathways. Such verification may also be of value since for the cluster-based selected peptides, a large fraction of the response is mediated by binders with relatively low predicted affinity (median of IC_50_: 217 nM). Accordingly, all 201 epitopes identified by the cluster-based approach (Subsets I, III and the BALB/c screen), the 6 responder peptides identified in Subsets II and IV as well as the 17 epitopes identified by the high affinity-based approach (Subset V and the BALB/c screen) were incubated with splenocytes (from C57BL/6 and BALB/c) supplemented with either anti-CD8 antibodies, anti-CD4 antibodies or a mixture of both antibodies, and subjected to the EliSpot assay (Figure 5). The complete information on the 222 responders of *F.tularensis* CTL, based on the analysis described above, is compiled in Table S5.

Without exception, anti-CD8 antibodies completely inhibited the IFNγ-induced response mediated by the tested peptides, while anti-CD4 antibodies did not alter the peptide-induced activity. We note that the presence of both anti-CD4 and anti-CD8 was needed to completely inhibit the killed LVS-mediated response. In conclusion, we unequivocally confirmed that all the 222 *F. tularensis* responder peptides, whether originating from the cluster-based approach (Subsets I, III) or the high affinity-based approach (Subset V), or the other subsets elicit a cytotoxic T-cell response and not a helper T-cell response.

## Discussion

Mapping the cellular immunome of a complex microbial genome, i.e. a bacterial pathogen, by identification of CTL epitopes, requires a rigorous filtering approach for down-selection of peptide candidates for experimental evaluation, and preferably an approach which enriches the extent of responders from the tested pool. In this regard, the present study further validates and expands the applicability of the cluster-based approach. The high extent of response obtained in our previous study [14] for peptides predicted to be MHC binders contained within regions rich in putative MHC binders (density of 1.0–1.4) is now shown to be attained also for peptide located in cluster regions of somewhat lower densities (0.8–1.0). It should be noted that even a density of 0.8–1.0 still corresponds to a substantial number of peptides per cluster region. Thus, a total of 43 novel CTL epitopes were discovered, constituting 10.7% of the 401 peptides evaluated from this subset of peptides (Subset III, Figure 2). The percent of responders will obviously decrease at much lower densities and indeed, predicted MHC binders chosen at random (Subset II) which have an average cluster density of 0.2–0.3 exhibit a 17–25 fold lower percentage of responders as compared to that obtained for cluster-based Subsets I and III (density 0.8–1.4). It should be noted that the absolute value of the density *per se* is consequential to the number of MHC alleles for which predictions are performed. Therefore, in cases of high diversity in MHC alleles, such as in humans, the criteria for cutoff of high density will probably be much higher because clusters are expected to contain much larger number of peptides per unit length.

Taken together, in the present and the previous studies 1641 peptides (Subsets I and III) located in high dense clusters were evaluated in C57BL/6, resulting in identification of a total of 170 novel CTL responders. Among these 170 8-11mer responder peptides, 44 peptides are “nested". This observation is the result of the operational definition of a cluster, where putative epitopes contained in a cluster are consecutive and may partially overlap. In the case of “nested" peptides, it is apparently possible that the longer peptide may be subjected to trimming processes, resulting in the stimulation of the same T-cell population as that stimulated by the embedded responder. Determining the actual MHC binders among the nested peptides is beyond the scope of the current study, but even if one considers only the 126 “unique" responders (to avoid redundancy in number of responders), the percentage of responders is 7.7%, which is still significantly higher (19-fold) than that observed for responders selected at random (Subset II).

**Figure 6:**
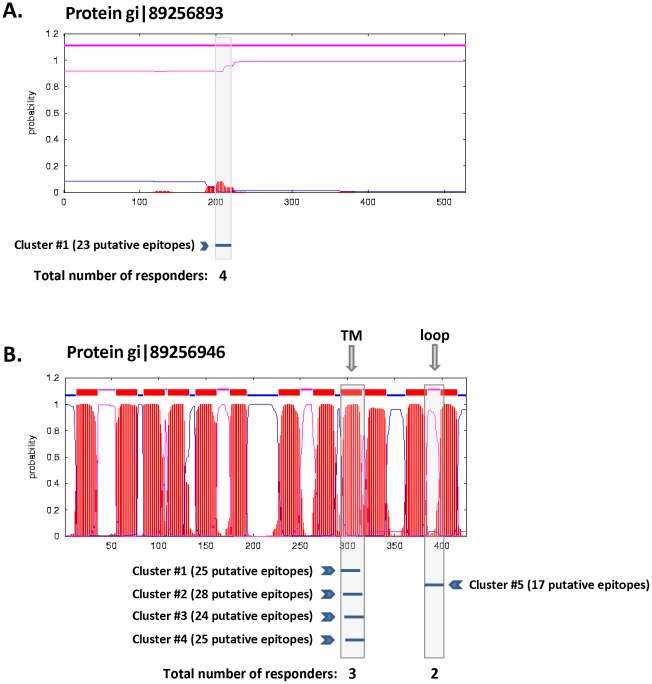
Distribution of clusters of CTL epitopes in a representative non-membranal and membrane-spanning protein and alignment with transmembrane α-helices and loops. Sample plots of the topological map (generated by TMHMM predictions) of two representative proteins. Red segments represent the probability of having a helical region, while the thin blue and pink lines represent inside and outside loops, respectively. In the lower part of every protein chart, a bar represents a cluster of predicted epitopes. Co-localization of the putative MHC binders with the predicted helix or loop region in the protein is marked by a grey box. (A) A sample of a non-membranal protein (gi|89256893) carrying a cluster at density 0.8, harboring 23 putative epitopes, four of which are a responders. This represents the 18% non-membranal proteins carrying high density clusters (see Figure 4). (B) A sample of a transmembrane protein (gi|89256946) carrying four clusters (clusters #1–4) within a α-helical transmembrane domain, (marked by “TM"), and an additional cluster (cluster #5) co-localized with an outside loop domain (marked by “loop") between two transmembranal regions. The total number of putative epitopes in clusters #1–4 is 102 and in cluster #5 is 17, which contain 3 and 2 responders, respectively. This sample protein provides an example for the high frequency of clusters in helical regions but also demonstrates the presence of high density clusters in loops (there are examples of other proteins, not shown, where clusters span both transmembrane and loop regions).

Investigation of the parental proteins of the responders with respect to their membranal characteristic (Figure 4) reveals a clear pattern of enrichment (70–80%) of membrane-integral proteins among proteins harboring highly dense epitope regions (Subset I as well as III) as compared to their prevalence in the whole genome (30%) or to proteins containing responders identified and selected at random (Subset II) or by high affinity (Subset V). The broadly immunogenic nature of transmembrane regions was also reported by others [19]. It is clear however that the factor of 2.5-fold increase in enrichment of membranal proteins (in Subsets I and III) would not account by itself for the high extent of response in the cluster-based approach (which is at least 17-fold). In depth inspection of cluster regions of Subset III within their parental proteins reveal that out of the 31 clusters, 5 are embedded in non-membranal protein (see a sample protein in Figure 6A), four of which contain CTL responders. The 26 remaining clusters are all included in transmembranal proteins. About 70% of these clusters are located in transmembrane helices while the remainders are mostly located in inter-connecting loops (Figure 6B). A similar observation was demonstrated previously for Subset I [14]. It is worth noting that the distribution of non-clustered epitopes between structural domains (e.g. peptides from Subset IV) is comparable to the distribution of clustered epitopes. Therefore it appears that the structural characteristics, i.e. transmembrane domains, are not a discriminatory characteristic, further supporting our argument that localization in such hydrophobic domains would not account by itself for the high extent of response in the cluster-based approach.

To further demonstrate the generality of the cluster-based approach, we extended the procedure which was carried out for Subsets I–V on C57BL/6 mice with Kb/Db allelic restriction, to BALB/c mice possessing Kd/Dd MHC alleles. Out of 262 peptides derived from highly dense clusters (densities of 0.8–1.4), 29 were found to elicit an immune response (11.1%), a success rate which is similar to that obtained in the C57BL/6 model (Table 1).

One of the main goals of the present study was to compare the cluster-based approach to the more conventional approach based on predicted binding affinities of putative epitopes to MHC molecules. In the latter, peptides are ranked according to their predicted affinity, and the top-ranking peptides are selected as the most likely immunogenic candidates. We have applied this approach to the genome of *F. tularensis* and selected a group of peptides predicted to be MHC binders with high affinity - IC_50_ values < = 10 nM. As expected, this approach yielded a high percent of responders - 9.8% in C57BL/6 and 7.4% in BALB/c (Table 1). The number of peptides selected from the cluster-based approach with IC_50_ values lesser than 10 nM is 25. Of the 170 responders resulting from the cluster-based approach, only 5 peptides have an IC_50_ value lesser than 10 nM. The latter were obviously included in the 418 peptides having an affinity of 10 nM and less, but were not part of the 92 peptides selected for evaluation, therefore no overlap could have been observed between responders identified by the cluster-based approach and responders identified by the high affinity-based approach. In this context we should mention that neither the cluster-based nor the high affinity-based screens were exhaustive (1641 out of 5729 and 92 out of 418 were tested, respectively, see Figure 2). In addition, had we selected putative epitopes solely on the basis of their predicted binding affinities (<10 nM), probably most of the 170 responders in C57BL/6 and the 29 in BALB/c would have been missed. All peptides selected on the basis of the high cluster density (0.8–1.4) and found to elicit an immune response have a range of predicted IC_50_ values of 5–988 nM with a median value of 217 nM. Therefore, even if we would have selected peptides with predicted affinities of <100 nM, almost 70% of the responders would have not been identified. However it is worth mentioning that although these peptides may be considered to have medium or relatively low predicted affinities it is possible that some of the peptides can be trimmed further and thus the resulting shorter peptides may have higher affinities, yet of the 130 peptides with predicted affinities above 100 nM, about 55% are short peptides (8 or 9mers). Note, that all of the responder peptides identified were shown in the present study to elicit IFNγ response which could be inhibited by anti-CD8 but not CD4 antibodies (Figure 5). Accordingly, the 201 cluster-based responder peptides and 17 high affinity based responder peptides are true CTL *F. tularensis* epitopes, validating again the reliability of the MHC prediction tools for selecting such T-cell epitopes [20], [21], [22], [23].

While no clear correlation exists between the intensity of the response of a particular epitope and its predicted IC_50_ values, we note that among the cluster-based peptides, there appears to be some correlation between the level of response and the average affinity (the geometric mean of predicted IC_50_ values being 183 nM for low-level responders and 160 nM and 117 nM for medium and high-level responders, respectively). Moreover, there appears to be an enrichment of peptides with higher predicted affinities concomitant with increase in the density [14]. Overall, the data demonstrate that the two approaches – high affinity based and cluster based - are not mutually exclusive but are rather complementary yielding largely different populations of CTL epitopes. The study reported here along with our previous study [14] provides a total of 2803 evaluated putative MHC I binders of which 222 are novel *F. tularensis* CTL epitopes, not documented before (as deduced from inspection of the data deposited in the comprehensive IEDB database, www.immuneepitope.org). This large body of data increases very significantly the knowledge on *F. tularensis* CTL epitopes.

Most if not all of the previous bacterial immunoinformatic analyses were limited to subsets of preselected antigens mostly on the basis of cellular location, or other functional characteristics or some documented immunogenicity of the parental antigens [12], [13], [24], [25]. The challenge of an unbiased, whole genomic immuno-analysis of a complex pathogen targeted for high-throughput analysis, as mentioned above, is mainly to balance between the very large number of predicted CTL epitope candidates and an experimental cost-effective screen that would yield a substantial fraction of the CTL-directed immune repertoire of the organism. The percentage range of CTL epitopes expected to reside in a bacterial proteome is still not well established and would probably depends on the pathogenesis of the organism and more specifically on the host-pathogen interactions, as well as on whether the disease is chronic or acute. For *F. tularensis* which is an intracellular obligatory pathogen causing an acute disease, we can be guided by the data obtained in our experiments from the percent responders of randomly selected predicted MHC binders (Subset II (0.4% responders) and Subset IV (2% responders); Figure 2). Accordingly, one can roughly estimate that out of all 90,879 peptides predicted as MHC binders in the *F. tularensis* genome (Fig. 1), there should be ∼1800 CTL epitopes or less. In our cluster-based screen, we tested 1641 peptides, out of 5729 peptides (Subsets I and III) mapped from highly dense cluster regions (densities 0.8–1.4) and identified 10.4% responders. Therefore one can deduce that the total number of responders out of the whole group of 5729 peptides would be ∼600. This number of epitopes is about a third of the above total estimated number of CTL epitopes in *F. tularensis*. While this surprisingly high percent of coverage of responders should be considered with great caution, it appears that by rationally selecting only a small fraction of the total predicted MHC binders one may recover a substantial portion of all expected responders in the bacterial genome. Considering the cluster-based approach success and power in filtering and enrichment of true positive responders in *F. tularensis*, we believe that this approach should be valuable and applicable for whole-genome, unbiased identification of CTL epitopes in other bacterial proteomes.

## Materials and Methods

### Ethics statement

This study was carried out in strict accordance with the recommendations in the Guide for the Care and Use of Laboratory Animals of the National Institute of Health. The protocol was approved by the Israel Institute for Biological Research Animal Care and Use Committee (Permit Number: IACUC-IIBR M-32-2011).

### Peptide synthesis

All peptides were synthesized by the 9-fluorenyl-methyloxycarbonyl (FMOC) chemistry and validated by mass spectrometry (Sigma, Israel). Peptides were adjusted to 500 µM stock solutions and stored at −20°C until use.

### Preparation of splenocytes

Groups of C57BL/6 or BALB/c mice were immunized with 10^2^ CFU LVS by the i.n route after anesthesia with Ketamine and Xylazine. Six weeks later mice were euthanized, spleens removed and splenocytes were prepared using a gentle MACS C-tube (Milteny, Germany) according to the manufacturer's instruction. The freshly prepared splenocytes were suspended in RPMI-1640 supplemented with 10% heat inactivated fetal calf serum and 1 mM of Pen-Strep, non-essential amino acids, 2 mM L-glutamine and sodium pyruvate. All tissue culture media and related reagents were from Biological Industries, Bet Haemek, Israel.

### IFNγ EliSpot assay

A single-cell suspension of fresh splenocytes (from either C57BL/6 or BALB/c) was seeded in EliSpot 96-well plates (MultiScreen Filter Plates, Millipore Ireland) at 10^6^ cells per well in complete RPMI medium containing 10 µM of each individual peptide or 10^7^ CFU/ml Formalin inactivated LVS as a positive control in duplicates. The frequency of epitope-specific T lymphocytes was determined after 24 hrs incubation at 37°C using eBioscience IFNγ EliSpot kits with strict adherence to manufacturer's instructions. A well was considered to be positive when it contained >10 spots and had at least twice the number of spots in negative control well. The background number of spots in negative control wells never exceeded five spots per well. Virtually all of the responses that were considered positive according to these criteria were also positive by the two-tailed non-parametric Mann–Whitney *U*-test (at *p*<0.05). Peptides which were shown to stimulate lymphocytes and induce IFNγ production are referred in the text as responders.

For determination of CD4 or CD8 T cell response, responder peptides at concentration of 10 µM were incubated with or without 10 µg/ml anti-mouse CD4 (Functional Grade Purified, clone GK1.5) or CD8 (Functional Grade Purified, clone 53-6.7) monoclonal antibodies (eBioscience, San Diego, USA) and tested for IFNγ production by EliSpot assay as mentioned above.

### Prediction of MHC class I putative binders

A total of 1754 *F. tularensis* ORF products (holarctica LVS strain, GenBank accession AM233362) were subjected to analysis for identification of putative CTL epitopes, by the MHC class I predictor NetMHC3.0 using artificial neural networks (ANN) [26]. Mouse MHC alleles were considered (H2-Kb, H2-Db, H2-Kd, H2-Dd). Predicted affinities (IC_50_, nM) for all 8, 9, 10, 11-mer peptides were computed by the NetMHC3.0 predictor.

### Cluster mapping and cluster density groups

A cluster of epitopes was defined as a polypeptide of up to 25 amino acids, containing at least 2 consecutive overlapping predicted binders. The density of a given cluster was designated as the number of predicted binders contained within the cluster divided by its length. Maximal density of all mapped clusters in the genome is 1.4. Clusters with a density equal or greater than 1 is defined throughout the paper as cluster density group 1.0–1.4. Clusters with densities 0.8 up to 1.0 (not including 1.0) is defined as cluster density group 0.8–1.0.

### Transmembranal helices and signal peptide predictions

Analysis of membrane protein topology was conducted by the program TMHMM v2.0 [27] for prediction of transmembranal helices based on hidden Markov model. Analysis of proteins for presence of a signal peptide domain was performed by the Signalp 3.0 server [28]. Proteins predicted to possess 10 or more predicted transmembranal domains (TM) and/or TMs spanning at least a third of the protein were assigned as membranal; Proteins predicted to possess less than 10 predicted TMs were assigned as partially membranal; proteins with no predicted transmembranal domains except signal peptide were assigned as “No TMs".

### Statistics

Statistical differences in EliSpot responses were determined using the two-tailed non-parametric Mann–Whitney *U*-test. Differences between number of responders obtained by the different selection approaches and between number of transmembrane and non-transmembrane proteins in the various subsets were determined using analysis of variance (ANOVA) and Student-Newman-Keuls tests. P-values< = 0.05 were considered as significant.

## Supporting Information

Table S1 List of 401 peptides selected from clusters of density 0.8 up to 1.0 (not including 1.0). The affinity provided is the IC_50_ value predicted for a particular responder sequence by the NetMHC3.0 program. The gi number and annotation of the source protein are according to the *F. tularensis* holarctica LVS sequence deposited at the NCBI (GenBank accession AM233362); (a) Responders are indicated by their magnitude of T-cell response as follows number of spots/million cells) is: L (Low): 5–20; M (medium) - 20–32; H (high) - 33 and above.
(PDF)

Table S2 List of 200 peptides contained in the 41 parental proteins of responder peptides, and selected from regions outside high density clusters (1.0–1.4) in these 41 proteins. The affinity provided is the IC_50_ value predicted for a particular responder sequence by the NetMHC3.0 program, and the threshold for binding affinity is 500 nM. The gi number and annotation of the source protein are according to the *F. tularensis* holarctica LVS sequence deposited at the NCBI (GenBank accession AM233362); (a) Responders are indicated by their magnitude of T-cell response as follows number of spots/million cells) is: L (Low): 5–20; M (medium) - 20–32; H (high) - 33 and above.
(PDF)

Table S3 List of 92 peptides selected from the high affinity-based approach, having IC50 values< = 10 nM. The affinity provided is the IC_50_ value predicted for a particular responder sequence by the NetMHC3.0 program. The gi number and annotation of the source protein are according to the *F. tularensis* holarctica LVS sequence deposited at the NCBI (GenBank accession AM233362); (a) Responders are indicated by their magnitude of T-cell response as follows number of spots/million cells) is: L (Low): 5–20; M (medium) - 20–32; H (high) - 33 and above.
(PDF)

Table S4 List of 370 peptides (262 selected by the cluster-based approach - indicated as “clu" and 108 selected by the affinity-based approach - indicated as “aff") tested in the BALB/c screen. The affinity provided is the IC_50_ value predicted for a particular responder sequence by the NetMHC3.0 program. The gi number and annotation of the source protein are according to the *F. tularensis* holarctica LVS sequence deposited at the NCBI (GenBank accession AM233362); (a) Responders are indicated by their magnitude of T-cell response as follows (number of spots/million cells) : L (Low): 5–20; M (medium) - 20–32; H (high) - 33 and above.
(PDF)

Table S5 The list of peptides provides the compiled data of responders from previously identified responders (Subsets I and II; [14]) and every responder listed in Tables S1, S2, S3, S4.
(PDF)

## References

[1] Hepburn MJ, Simpson AJ (2008). Tularemia: current diagnosis and treatment options.. Expert Rev Anti Infect Ther.

[2] Sjostedt A (2006). Intracellular survival mechanisms of *Francisella tularensis*, a stealth pathogen.. Microbes Infect.

[3] Titball RW, Petrosino JF (2007). *Francisella tularensis* genomics and proteomics.. Ann N Y Acad Sci.

[4] Barry EM, Cole LE, Santiago AE (2009). Vaccines against Tularemia.. Hum Vaccin.

[5] Conlan JW (2011). Tularemia vaccines: recent developments and remaining hurdles.. Future Microbiol.

[6] Conlan W, Sjostedt A, Shafferman A, Ordentlich A, Velan B (2010). Novel live vaccine candidates against airborne *Francisella tularensis*;.

[7] Griffin KF, Oyston PC, Titball RW (2007). *Francisella tularensis* vaccines.. FEMS Immunol Med Microbiol.

[8] Mann BJ, Ark NM (2009). Rationally designed Tularemia vaccines.. Expert Rev Vaccines.

[9] McMurry JA, Moise L, Gregory SH, De Groot AS (2007). Tularemia vaccines - an overview.. Med Health R I.

[10] Oyston PC (2009). *Francisella tularensis* vaccines.. Vaccine.

[11] Conlan JW, Sjostedt A, North RJ (1994). CD4+ and CD8+ T-cell-dependent and -independent host defense mechanisms can operate to control and resolve primary and secondary *Francisella tularensis* LVS infection in mice.. Infect Immun.

[12] Gregory SH, Mott S, Phung J, Lee J, Moise L (2009). Epitope-based vaccination against pneumonic Tularemia.. Vaccine.

[13] McMurry JA, Gregory SH, Moise L, Rivera D, Buus S (2007). Diversity of *Francisella tularensis* Schu4 antigens recognized by T lymphocytes after natural infections in humans: identification of candidate epitopes for inclusion in a rationally designed Tularemia vaccine.. Vaccine.

[14] Zvi A, Rotem S, Bar-Haim E, Cohen O, Shafferman A (2011). Whole-genome immunoinformatic analysis of *F. tularensis*: predicted CTL epitopes clustered in hotspots are prone to elicit a T-cell response.. PLoS One.

[15] Lund O, Nascimento EJ, Maciel M, Nielsen M, Larsen MV (2011). Human leukocyte antigen (HLA) class I restricted epitope discovery in yellow fewer and dengue viruses: importance of HLA binding strength.. PLoS One.

[16] Moutaftsi M, Peters B, Pasquetto V, Tscharke DC, Sidney J (2006). A consensus epitope prediction approach identifies the breadth of murine T(CD8+)-cell responses to vaccinia virus.. Nat Biotechnol.

[17] Sette A, Vitiello A, Reherman B, Fowler P, Nayersina R (1994). The relationship between class I binding affinity and immunogenicity of potential cytotoxic T cell epitopes.. J Immunol.

[18] Yewdell JW, Bennink JR (1999). Immunodominance in major histocompatibility complex class I-restricted T lymphocyte responses.. Annu Rev Immunol.

[19] Kovjazin R, Volovitz I, Daon Y, Vider-Shalit T, Azran R (2011). Signal peptides and trans-membrane regions are broadly immunogenic and have high CD8+ T cell epitope densities: Implications for vaccine development.. Mol Immunol.

[20] Larsen MV, Lundegaard C, Lamberth K, Buus S, Lund O (2007). Large-scale validation of methods for cytotoxic T-lymphocyte epitope prediction.. BMC Bioinformatics.

[21] Lin HH, Ray S, Tongchusak S, Reinherz EL, Brusic V (2008). Evaluation of MHC class I peptide binding prediction servers: applications for vaccine research.. BMC Immunol.

[22] Lundegaard C, Lund O, Nielsen M (2011). Prediction of epitopes using neural network based methods.. J Immunol Methods.

[23] Lundegaard C, Lund O, Nielsen M (2012). Predictions versus high-throughput experiments in T-cell epitope discovery: competition or synergy?. Expert Rev Vaccines.

[24] McMurry JA, Kimball S, Lee JH, Rivera D, Martin W (2007). Epitope-driven TB vaccine development: a streamlined approach using immuno-informatics, ELISpot assays, and HLA transgenic mice.. Curr Mol Med.

[25] Moise L, McMurry JA, Pappo J, Lee DS, Moss SF (2008). Identification of genome-derived vaccine candidates conserved between human and mouse-adapted strains of *H. pylori*.. Hum Vaccin.

[26] Lundegaard C, Lamberth K, Harndahl M, Buus S, Lund O (2008). NetMHC-3.0: accurate web accessible predictions of human, mouse and monkey MHC class I affinities for peptides of length 8–11.. Nucleic Acids Res.

[27] Krogh A, Larsson B, von Heijne G, Sonnhammer EL (2001). Predicting transmembrane protein topology with a hidden Markov model: application to complete genomes.. J Mol Biol.

[28] Bendtsen JD, Nielsen H, von Heijne G, Brunak S (2004). Improved prediction of signal peptides: SignalP 3.0.. J Mol Biol.

