# Supplementary material for: Clusters versus Affinity-Based Approaches in F. tularensis Whole Genome Search of CTL Epitopes
Source: PLoS One. 2012 May 1;7(5):e36440. doi: 10.1371/journal.pone.0036440 (PMC3341354; doi:10.1371/journal.pone.0036440)
Supplement: Table S1 — List of 401 peptides selected from clusters of density 0.8 up to 1.0 (not including 1.0). The affinity provided is the IC50 value predicted for a particular responder sequence by the NetMHC3.0 program. The gi number and annotation of the source protein are according to the F. tularensis holarctica LVS sequence deposited at the NCBI (GenBank accession AM233362); (a) Responders are indicated by their magnitude of T-cell response as follows number of spots/million cells) is: L (Low): 5–20; M (medium) - 20–32; H (high) - 33 and above. (PDF) [file pone.0036440.s001.pdf]

Table S1: Compilation of data for cluster-based selected putative MHC binders (density 0.8-1.0, Subset III)

Sequence	Length	Predicted allele	Affinity (IC_{50}, nM)	Protein gi#	Responder[(a)]
SYAKINLFLHI	11	H-2_Kd	72	89255588	
SYAKINLFL	9	H-2_Kd	110	89255588	
YAKINLFLHI	10	H-2_Db	319	89255588	
YAKINLFLHIL	11	H-2_Db	410	89255588	
YAKINLFL	8	H-2_Db	296	89255588	
AKINLFLHIL	10	H-2_Kb	487	89255588	
KKYYSYAKINL	11	H-2_Kb	121	89255588	
KKYYSYAKI	9	H-2_Kb	425	89255588	
KYYSYAKINL	10	H-2_Kb	379	89255588	
KYYSYAKI	8	H-2_Kb	18	89255588	
YSYAKINLFL	10	H-2_Kb	217	89255588	
YSYAKINL	8	H-2_Kb	226	89255588	
ALMNWVIPL	9	H-2_Kb	334	89255626	L
HGVIMIFFVAM	11	H-2_Kb	493	89255626	
GVIMIFFVAM	10	H-2_Kb	369	89255626	
VIMIFFVAMPL	11	H-2_Kb	53	89255626	
VIMIFFVAM	9	H-2_Kb	23	89255626	
IMIFFVAMPL	10	H-2_Kb	24	89255626	
IMIFFVAMPLI	11	H-2_Kb	254	89255626	
IMIFFVAM	8	H-2_Kb	113	89255626	
IMIFFVAMP	9	H-2_Kb	251	89255626	
MIFFVAMPL	9	H-2_Kb	133	89255626	
IFFVAMPL	8	H-2_Kb	302	89255626	
VAMPLIFALM	10	H-2_Kb	144	89255626	
VAMPLIFAL	9	H-2_Kb	127	89255626	
AMPLIFALM	9	H-2_Kb	105	89255626	
FALMNWVIPL	10	H-2_Db	11	89255626	
FALMNWVI	8	H-2_Db	9	89255626	
FALMNWVIP	9	H-2_Db	94	89255626	
VLYYLVNLLTL	11	H-2_Kb	30	89255629	L
CLVLYYLVNL	10	H-2_Kb	197	89255629	
CLVLYYLVNLL	11	H-2_Kb	169	89255629	
LVLYYLVNLL	10	H-2_Kb	37	89255629	
LVLYYLVNL	9	H-2_Kb	37	89255629	
VLYYLVNL	8	H-2_Kb	117	89255629	
VLYYLVNLL	9	H-2_Kb	19	89255629	
LYYLVNLLTL	10	H-2_Kb	237	89255629	
LYYLVNLL	8	H-2_Kb	228	89255629	
YYLVNLLTLWI	11	H-2_Db	40	89255629	

YYLVNLLTL	9	H-2_Db	7	89255629	
TILLILSCL	9	H-2_Kb	445	89255629	
LILSCLVLYYL	11	H-2_Kb	429	89255629	
SCLVLYYLVNL	11	H-2_Kb	320	89255629	
SCLVLYYL	8	H-2_Db	434	89255629	
AAISFLYLLAV	11	H-2_Kb	921	89255755	
AAISFLYL	8	H-2_Db	240	89255755	
AAISFLYLL	9	H-2_Kb	87	89255755	
ISFLYLLAVA	10	H-2_Kb	760	89255755	
ISFLYLLAVAL	11	H-2_Kb	13	89255755	
ISFLYLLA	8	H-2_Kb	175	89255755	
ISFLYLLAV	9	H-2_Kb	143	89255755	
SFLYLLAVAL	10	H-2_Kb	151	89255755	
FLYLLAVALRL	11	H-2_Kb	502	89255755	
FLYLLAVAL	9	H-2_Kb	112	89255755	
LYLLAVALRL	10	H-2_Kd	952	89255755	
LYLLAVAL	8	H-2_Kd	78	89255755	
VALRLAKFDTM	11	H-2_Db	575	89255755	
LRLAKFDTM	9	H-2_Kb	324	89255755	
ANISLVVSL	9	H-2_Kb	149	89255770	
ISLVVSLTFL	10	H-2_Db	202	89255770	
SLVVSLTFL	9	H-2_Db	95	89255770	
LVVSLTFLTTL	11	H-2_Kb	284	89255770	
VVSLTFLTTL	10	H-2_Kb	337	89255770	
VSLTFLTTLL	10	H-2_Kb	60	89255770	
VSLTFLTT	8	H-2_Kb	270	89255770	
VSLTFLTTL	9	H-2_Db	223	89255770	
LTFLTTLLAPI	11	H-2_Kb	307	89255770	
LTFLTTLL	8	H-2_Kb	462	89255770	
TFLTTLLAPI	10	H-2_Kd	101	89255770	
TTLLAPIFMPL	11	H-2_Kb	80	89255770	
TTLLAPIFM	9	H-2_Db	375	89255770	
TLLAPIFMPL	10	H-2_Kb	395	89255770	
LAPIFMPL	8	H-2_Kb	133	89255770	
LAPIFMPLI	9	H-2_Kb	275	89255770	
VWSIIYISL	9	H-2_Kb	329	89255790	L
WSIIYISL	8	H-2_Kb	21	89255790	L
SIIYISLGYL	10	H-2_Kb	482	89255790	L
SSFVWSIIYI	10	H-2_Db	335	89255790	
SFVWSIIYISL	11	H-2_Kb	222	89255790	
FVWSIIYISL	10	H-2_Kb	270	89255790	
WSIIYISLGYL	11	H-2_Db	365	89255790	
IIYISLGYL	9	H-2_Kb	71	89255790	
IYISLGYL	8	H-2_Kb	301	89255790	
ISLGYLFGNTI	11	H-2_Kb	252	89255790	
LGYLFGNTIL	10	H-2_Kb	54	89255790	
LGYLFGNT	8	H-2_Kb	32	89255790	

LGYLFGNTI	9	H-2_Kb	62	89255790	
GYLFGNTI	8	H-2_Kd	12	89255790	
GYLFGNTIL	9	H-2_Kd	6	89255790	
CVSNNISSPTM	11	H-2_Kb	434	89255853	
VSNNISSPTM	10	H-2_Kb	686	89255853	
PTMKKYYYNTL	11	H-2_Kb	102	89255853	
TMKKYYYNTL	10	H-2_Kb	36	89255853	
MKKYYYNTL	9	H-2_Kb	45	89255853	
KKYYYNTLIIL	11	H-2_Kb	163	89255853	
KKYYYNTL	8	H-2_Kb	218	89255853	
KYYYNTLIIL	10	H-2_Db	803	89255853	
KYYYNTLIILL	11	H-2_Kb	156	89255853	
KYYYNTLI	8	H-2_Kd	90	89255853	
KYYYNTLII	9	H-2_Db	301	89255853	
YYYNTLIILL	10	H-2_Kb	243	89255853	
YYYNTLIILLL	11	H-2_Kb	454	89255853	
YYYNTLII	8	H-2_Kd	696	89255853	
YYYNTLIIL	9	H-2_Kb	319	89255853	
YYNTLIILLL	10	H-2_Kd	479	89255853	
YYNTLIIL	8	H-2_Kd	86	89255853	
YYNTLIILL	9	H-2_Kd	214	89255853	
KNYQNLVM	8	H-2_Db	330	89255900	M
TISYYKNYQNL	11	H-2_Kb	255	89255900	
ISYYKNYQNL	10	H-2_Kb	10	89255900	
ISYYKNYQNLV	11	H-2_Kb	75	89255900	
SYYKNYQNLV	10	H-2_Kb	307	89255900	
SYYKNYQNLVM	11	H-2_Db	253	89255900	
SYYKNYQNL	9	H-2_Db	277	89255900	
LVATNYIVL	9	H-2_Db	54	89255968	
VATNYIVL	8	H-2_Kb	527	89255968	
TNYIVLTSV	9	H-2_Kb	479	89255968	
NYIVLTSVSWI	11	H-2_Kd	84	89255968	
NYIVLTSV	8	H-2_Kd	33	89255968	
TSVSWIFYLL	10	H-2_Kb	159	89255968	
TSVSWIFYLLM	11	H-2_Kb	143	89255968	
TSVSWIFYL	9	H-2_Kb	135	89255968	
SVSWIFYLLM	10	H-2_Kb	631	89255968	
SVSWIFYLL	9	H-2_Kb	706	89255968	
VSWIFYLLMFL	11	H-2_Kb	58	89255968	
VSWIFYLL	8	H-2_Kb	6	89255968	
VSWIFYLLM	9	H-2_Kb	38	89255968	
SWIFYLLMFL	10	H-2_Kb	542	89255968	
WIFYLLMFL	9	H-2_Kb	432	89255968	
IFYLLMFLIAV	11	H-2_Kb	430	89255968	
IFYLLMFL	8	H-2_Kb	385	89255968	
FYLLMFLIAV	10	H-2_Kd	80	89255968	
FYLLMFLI	8	H-2_Db	832	89255968	

LTYNYGYA	8	H-2_Kb	120	89255979	M
VLYFMGACI	9	H-2_Kb	865	89255979	L
LYFMGACI	8	H-2_Kd	57	89255979	L
LTYNYGYAPV	10	H-2_Kb	116	89255979	
LTYNYGYAPVL	11	H-2_Kb	43	89255979	
LTYNYGYAP	9	H-2_Kb	561	89255979	
TYNYGYAPVL	10	H-2_Kd	450	89255979	
TYNYGYAPV	9	H-2_Kd	191	89255979	
YNYGYAPV	8	H-2_Kb	5	89255979	
YNYGYAPVL	9	H-2_Kb	187	89255979	
NYGYAPVLYF	10	H-2_Dd	831	89255979	
YGYAPVLYFM	10	H-2_Db	842	89255979	
GYAPVLYFM	9	H-2_Kd	755	89255979	
YAPVLYFM	8	H-2_Db	19	89255979	
LYFMGACIVV	10	H-2_Kd	508	89255979	
LYFMGACIV	9	H-2_Kd	285	89255979	
KVLVFFSSM	9	H-2_Kb	127	89256021	L
VLVFFSSMLSL	11	H-2_Kb	313	89256021	L
VFFSSMLSL	9	H-2_Kb	448	89256021	L
VAFVFITFIL	10	H-2_Kb	72	89256047	
VAFVFITFILL	11	H-2_Kb	32	89256047	
VAFVFITF	8	H-2_Kb	183	89256047	
VAFVFITFI	9	H-2_Kb	283	89256047	
FVFITFILLFI	11	H-2_Db	955	89256047	
FVFITFILL	9	H-2_Kb	135	89256047	
VFITFILL	8	H-2_Kb	206	89256047	
ITFILLFILI	10	H-2_Kb	280	89256047	
ITFILLFILIL	11	H-2_Kb	82	89256047	
ITFILLFIL	9	H-2_Kb	104	89256047	
TFILLFILIL	10	H-2_Kb	836	89256047	
FILLFILI	8	H-2_Kb	722	89256047	
FILLFILIL	9	H-2_Kb	436	89256047	
SILLYSLTTL	10	H-2_Kb	109	89256177	L
SILLYSLTTLL	11	H-2_Kb	217	89256177	L
ILLYSLTTLL	10	H-2_Kb	221	89256177	L
ILLYSLTTL	9	H-2_Kb	110	89256177	M
LLYSLTTLL	9	H-2_Kb	141	89256177	H
LLYSLTTL	8	H-2_Kb	693	89256177	
TTLLCIFVNNI	11	H-2_Kb	720	89256177	
CIFVNNIYIFM	11	H-2_Db	193	89256177	
CIFVNNIYI	9	H-2_Db	52	89256177	
IFVNNIYIFM	10	H-2_Db	996	89256177	
IFVNNIYI	8	H-2_Db	873	89256177	
KGYNYGNYTLL	11	H-2_Kb	63	89256197	L
FKGYNYGNYTL	11	H-2_Dd	931	89256197	
KGYNYGNYTL	10	H-2_Kb	102	89256197	
KGYNYGNY	8	H-2_Kb	88	89256197	

GYNYGNYTLL	10	H-2_Dd	462	89256197	
GYNYGNYTL	9	H-2_Dd	159	89256197	
YNYGNYTLLL	10	H-2_Kb	241	89256197	
YNYGNYTL	8	H-2_Db	493	89256197	
YNYGNYTLL	9	H-2_Db	126	89256197	
NYTLLLSGVI	10	H-2_Kd	542	89256197	
NYTLLLSGV	9	H-2_Kd	761	89256197	
YTLLLSGVIM	10	H-2_Db	703	89256197	
YTLLLSGVIML	11	H-2_Db	911	89256197	
SGVIMLVIL	9	H-2_Kb	228	89256197	
VIMLVILVQL	10	H-2_Kb	413	89256197	
IMLVILVQL	9	H-2_Kb	149	89256197	
ISINLGSLL	9	H-2_Kb	308	89256276	L
SLLAYLVAPSL	11	H-2_Kb	94	89256276	L
LLAYLVAPSL	10	H-2_Kb	305	89256276	H
LAYLVAPSL	9	H-2_Kb	13	89256276	L
LGAFLLSMIAM	11	H-2_Kb	253	89256315	
GAFLLSMIAM	10	H-2_Kb	264	89256315	
AFLLSMIAM	9	H-2_Kb	976	89256315	
FLLSMIAMTL	10	H-2_Kb	811	89256315	
LSMIAMTLLTM	11	H-2_Db	441	89256315	
LSMIAMTLL	9	H-2_Kb	440	89256315	
SMIAMTLLTM	10	H-2_Db	60	89256315	
SMIAMTLL	8	H-2_Db	487	89256315	
IAMTLLTMFL	10	H-2_Db	189	89256315	
IAMTLLTM	8	H-2_Db	93	89256315	
MTLLTMFLQYI	11	H-2_Db	850	89256315	
MTLLTMFL	8	H-2_Kb	794	89256315	
LTMFLQYIGI	10	H-2_Kb	745	89256315	
TMFLQYIGIFL	11	H-2_Db	808	89256315	
TMFLQYIGI	9	H-2_Kb	75	89256315	
SFFNYFKYMGM	11	H-2_Kb	103	89256429	H
SFFNYFKYM	9	H-2_Kb	101	89256429	H
FNYFKYMGM	9	H-2_Kb	42	89256429	H
FKYMGMTLLAL	11	H-2_Kb	238	89256429	L
TITQYFILL	9	H-2_Kb	134	89256432	L
FILLNQYYQF	10	H-2_Db	328	89256432	L
FILLNQYYQFL	11	H-2_Db	13	89256432	L
ILLNQYYQFL	10	H-2_Kb	115	89256432	L
LNQYYQFL	8	H-2_Kb	391	89256432	L
YYQFLIQL	8	H-2_Kd	232	89256432	L
FVYIFGFISAL	11	H-2_Kb	49	89256438	
FVYIFGFI	8	H-2_Kb	178	89256438	
VYIFGFISAL	10	H-2_Kb	93	89256438	
VYIFGFISALI	11	H-2_Kd	422	89256438	
YIFGFISAL	9	H-2_Kb	141	89256438	
GFISALIYVYL	11	H-2_Kd	747	89256438	

ISALIYVYLL	10	H-2_Kb	112	89256438	
ISALIYVYL	9	H-2_Db	833	89256438	
SALIYVYL	8	H-2_Db	58	89256438	
SALIYVYLL	9	H-2_Db	248	89256438	
LIYVYLLY	8	H-2_Kb	379	89256438	
IYVYLLYSWEL	11	H-2_Kb	749	89256438	
YVYLLYSWEL	10	H-2_Db	674	89256438	
VYLLYSWEL	9	H-2_Kb	917	89256438	
NSYIINYKL	9	H-2_Kb	667	89256494	M
NSYIINYKLL	10	H-2_Kb	162	89256494	
SYIINYKLLNI	11	H-2_Db	548	89256494	
SYIINYKL	8	H-2_Db	532	89256494	
SYIINYKLL	9	H-2_Db	319	89256494	
YIINYKLL	8	H-2_Kb	41	89256494	
INYKLLNINL	10	H-2_Kb	41	89256494	
INYKLLNINLI	11	H-2_Kb	94	89256494	
INYKLLNI	8	H-2_Kb	70	89256494	
YKLLNINL	8	H-2_Db	669	89256494	
YKLLNINLI	9	H-2_Db	187	89256494	
LNINLINTQL	10	H-2_Kb	386	89256494	
INLINTQL	8	H-2_Kb	903	89256494	
INTQLKNTKIL	11	H-2_Kb	794	89256494	
TQLKNTKIL	9	H-2_Db	839	89256494	
LSSYLNARIFL	11	H-2_Kb	941	89256679	
SSYLNARIFL	10	H-2_Db	27	89256679	
SSYLNARIFLL	11	H-2_Db	110	89256679	
SSYLNARI	8	H-2_Db	257	89256679	
SYLNARIFLL	10	H-2_Kb	616	89256679	
SYLNARIFL	9	H-2_Kb	976	89256679	
IFLLAFIFVIL	11	H-2_Kb	395	89256679	
FLLAFIFVIL	10	H-2_Kb	718	89256679	
LAFIFVILVV	10	H-2_Kb	702	89256679	
LAFIFVILVVL	11	H-2_Kb	45	89256679	
LAFIFVIL	8	H-2_Kb	164	89256679	
LAFIFVILV	9	H-2_Kb	302	89256679	
FIFVILVVL	9	H-2_Kb	961	89256679	
AIMAMFESI	9	H-2_Kb	419	89256785	L
NGYISGAFIAL	11	H-2_Kb	149	89256785	
GYISGAFIAL	10	H-2_Kd	16	89256785	
GYISGAFIALI	11	H-2_Kd	55	89256785	
GYISGAFI	8	H-2_Kd	17	89256785	
ISGAFIALIFL	11	H-2_Kb	278	89256785	
ISGAFIAL	8	H-2_Kb	154	89256785	
ISGAFIALI	9	H-2_Kb	836	89256785	
SGAFIALIFL	10	H-2_Kb	965	89256785	
GAFIALIFL	9	H-2_Kb	670	89256785	
IALIFLAIMAM	11	H-2_Kb	27	89256785	

IALIFLAI	8	H-2_Kb	935	89256785	
IALIFLAIM	9	H-2_Kb	31	89256785	
ALIFLAIMAM	10	H-2_Kb	531	89256785	
LIFLAIMAM	9	H-2_Kb	125	89256785	
LAIMAMFESI	10	H-2_Kb	419	89256785	
LAIMAMFESIM	11	H-2_Kb	478	89256785	
AIMAMFESIM	10	H-2_Kb	441	89256785	
IMAMFESIM	9	H-2_Kb	365	89256785	
VLLSIMSYYLL	11	H-2_Kb	429	89256893	L
LSIMSYYLL	9	H-2_Db	61	89256893	L
SIMSYYLL	8	H-2_Kb	16	89256893	H
SIMSYYLLL	9	H-2_Kb	124	89256893	M
YTITSYQNAL	10	H-2_Db	270	89256946	L
VIIFLLVCL	9	H-2_Kb	167	89256946	L
VSIVNVTL	8	H-2_Db	98	89256968	L
VNVTLVPLL	9	H-2_Kb	620	89256968	L
VSIVNVTLVPL	11	H-2_Db	18	89256968	
VSIVNVTLV	9	H-2_Db	30	89256968	
SIVNVTLVPL	10	H-2_Kb	481	89256968	
SIVNVTLVPLL	11	H-2_Kb	936	89256968	
VNVTLVPL	8	H-2_Kb	133	89256968	
VTLVPLLILSL	11	H-2_Kb	279	89256968	
TLVPLLILSL	10	H-2_Kb	942	89256968	
LVPLLILSL	9	H-2_Kb	85	89256968	
LLILSLWYYL	10	H-2_Db	370	89256968	
LILSLWYYL	9	H-2_Kb	282	89256968	
LSLWYYLYGDI	11	H-2_Kb	261	89256968	
LSLWYYLY	8	H-2_Kb	845	89256968	
LSLWYYLYG	9	H-2_Kb	855	89256968	
LWYYLYGDI	9	H-2_Kb	573	89256968	
WYYLYGDI	8	H-2_Kb	992	89256968	
LSFTYVLASL	10	H-2_Kb	35	89256999	L
HLLLYFANIL	10	H-2_Kb	749	89256999	
LLLYFANI	8	H-2_Kb	899	89256999	
LLLYFANIL	9	H-2_Kb	100	89256999	
LLYFANILISP	11	H-2_Kb	816	89256999	
LYFANILISPL	11	H-2_Kb	118	89256999	
LYFANILI	8	H-2_Kd	702	89256999	
FANILISPL	9	H-2_Kb	701	89256999	
ANILISPL	8	H-2_Kb	968	89256999	
ISPLSFTYVL	10	H-2_Db	82	89256999	
ISPLSFTYV	9	H-2_Db	75	89256999	
SPLSFTYVL	9	H-2_Kb	428	89256999	
LSFTYVLASLL	11	H-2_Kb	145	89256999	
LSFTYVLA	8	H-2_Kb	155	89256999	
SFTYVLASL	9	H-2_Kb	895	89256999	
FTYVLASLLL	10	H-2_Kb	628	89256999	

FTYVLASL	8	H-2_Kb	11	89256999
FTYVLASLL	9	H-2_Kb	337	89256999
TYVLASLL	8	H-2_Kd	113	89256999
AKVLVFFSSM	10	H-2_Kb	260	89256021
KVLVFFSSML	10	H-2_Kb	386	89256021
VLVFFSSM	8	H-2_Kb	398	89256021
VLVFFSSML	9	H-2_Kb	345	89256021
LVFFSSMLSL	10	H-2_Kb	135	89256021
FFSSMLSLV	9	H-2_Kd	410	89256021
SSMLSLVDEL	10	H-2_Db	52	89256021
SMLSLVDEL	9	H-2_Db	192	89256021
ITVIKYSILL	10	H-2_Kb	140	89256177
ITVIKYSIL	9	H-2_Kb	113	89256177
TVIKYSIL	8	H-2_Kb	30	89256177
TVIKYSILL	9	H-2_Kb	210	89256177
VIKYSILLYSL	11	H-2_Kb	468	89256177
IKYSILLYSL	10	H-2_Kb	30	89256177
KYSILLYSL	9	H-2_Kd	233	89256177
YSILLYSLTTL	11	H-2_Db	32	89256177
YSILLYSL	8	H-2_Db	87	89256177
LYSLTTLL	8	H-2_Kd	298	89256177
YSLTTLLCIFV	11	H-2_Db	36	89256177
YSLTTLLCI	9	H-2_Db	14	89256177
SGMTMFYISI	10	H-2_Db	461	89256276
SGMTMFYI	8	H-2_Db	129	89256276
MTMFYISINL	10	H-2_Kb	196	89256276
MTMFYISI	8	H-2_Kb	102	89256276
TMFYISINL	9	H-2_Kb	52	89256276
MFYISINLGSL	11	H-2_Kb	163	89256276
MFYISINL	8	H-2_Kb	282	89256276
FYISINLGSL	10	H-2_Kd	5	89256276
FYISINLGSLL	11	H-2_Kd	10	89256276
ISINLGSL	8	H-2_Kb	71	89256276
INLGSLLAYL	10	H-2_Kb	303	89256276
LAYLVAPSLI	10	H-2_Kb	301	89256276
AYLVAPSL	8	H-2_Kd	80	89256276
AYLVAPSLI	9	H-2_Kd	41	89256276
ISFFNYFKYM	10	H-2_Db	139	89256429
FNYFKYMGMTL	11	H-2_Kb	49	89256429
NYFKYMGMTLL	11	H-2_Kd	144	89256429
NYFKYMGM	8	H-2_Kb	226	89256429
FKYMGMTLL	9	H-2_Kb	402	89256429
KYMGMTLLAL	10	H-2_Kd	20	89256429
KYMGMTLL	8	H-2_Kd	7	89256429
YMGMTLLAL	9	H-2_Db	190	89256429
TTITQYFILL	10	H-2_Kb	145	89256432
TTITQYFI	8	H-2_Db	192	89256432

TTITQYFIL	9	H-2_Kb	408	89256432
ITQYFILL	8	H-2_Kb	124	89256432
FILLNQYYQ	9	H-2_Db	121	89256432
LNQYYQFLIQL	11	H-2_Kb	323	89256432
NQYYQFLIQL	10	H-2_Kb	41	89256432
QYYQFLIQL	9	H-2_Kb	40	89256432
FVLLPIIINQL	11	H-2_Kb	315	89256861
FVLLPIII	8	H-2_Db	462	89256861
VLLPIIINQL	10	H-2_Kb	317	89256861
LLPIIINQL	9	H-2_Kb	286	89256861
KVLLSIMSYYL	11	H-2_Db	121	89256893
VLLSIMSYYL	10	H-2_Kb	494	89256893
LSIMSYYLLL	10	H-2_Db	260	89256893
LSIMSYYL	8	H-2_Db	49	89256893
MSYYLLLNDI	10	H-2_Kb	110	89256893
SYYLLLNDIGV	11	H-2_Kd	492	89256893
SYYLLLNDI	9	H-2_Kb	404	89256893
YYLLLNDIGV	10	H-2_Kd	492	89256893
YYLLLNDI	8	H-2_Kd	19	89256893
IGVFYCAV	8	H-2_Kb	116	89256893
IPLAFIILLVL	11	H-2_Kb	458	89256896
LAFIILLVL	9	H-2_Kb	206	89256896
IYTITSYQNAL	11	H-2_Kd	299	89256946
TITSYQNAL	9	H-2_Kb	173	89256946
TSYQNALDMV	10	H-2_Db	480	89256946
TSYQNALDMVI	11	H-2_Db	178	89256946
TSYQNALDM	9	H-2_Db	44	89256946
SYQNALDMVI	10	H-2_Kd	37	89256946
SYQNALDMVII	11	H-2_Kd	99	89256946
SYQNALDMV	9	H-2_Kd	87	89256946
MVIIFLLVCL	10	H-2_Kb	306	89256946
MVIIFLLV	8	H-2_Kb	462	89256946
IIFLLVCL	8	H-2_Kb	203	89256946
