# Supplementary material for: Clusters versus Affinity-Based Approaches in F. tularensis Whole Genome Search of CTL Epitopes
Source: PLoS One. 2012 May 1;7(5):e36440. doi: 10.1371/journal.pone.0036440 (PMC3341354; doi:10.1371/journal.pone.0036440)
Supplement: Table S2 — List of 200 peptides contained in the 41 parental proteins of responder peptides, and selected from regions outside high density clusters (1.0–1.4) in these 41 proteins. The affinity provided is the IC50 value predicted for a particular responder sequence by the NetMHC3.0 program, and the threshold for binding affinity is 500 nM. The gi number and annotation of the source protein are according to the F. tularensis holarctica LVS sequence deposited at the NCBI (GenBank accession AM233362); (a) Responders are indicated by their magnitude of T-cell response as follows number of spots/million cells) is: L (Low): 5–20; M (medium) - 20–32; H (high) - 33 and above. (PDF) [file pone.0036440.s002.pdf]

Table S2: Compilation of data for putative MHC binders selected from the 41 parental proteins (Subset IV)

Sequence	Length	Predicted allele	Affinity (IC_{50}, nM)	Protein gi#	Responder[(a)]
LSPNQPILF	9	H-2_Dd	472	89255563	L
SAILNSKADI	10	H-2_Db	61	89255563	L
LNIQDIISQL	10	H-2_Kb	356	89255563	
SSDLNKTII	9	H-2_Db	136	89255563	
TIIKQLNKL	9	H-2_Kb	206	89255563	
SRYIGYNNV	9	H-2_Kb	460	89255563	
RYIGYNNV	8	H-2_Kb	169	89255563	
RYIGYNNVL	9	H-2_Kd	120	89255563	
GYNNVLQKQEI	11	H-2_Kd	54	89255563	
KQYFKHNSL	9	H-2_Kb	19	89255563	
SYTKNVSSGV	10	H-2_Kd	80	89255563	
IKIQFHDPL	9	H-2_Kb	30	89255563	
WTKHKPSYI	9	H-2_Dd	129	89255563	
ILYAIEIAKEM	11	H-2_Kb	378	89255563	
LYLTMQLI	8	H-2_Kd	14	89255570	
LGFIPQIGFL	10	H-2_Kb	356	89255570	
FFPHNGATVI	10	H-2_Dd	172	89255570	
VIFLLYLAGI	10	H-2_Kb	198	89255570	
GYIIKFTFL	9	H-2_Kd	18	89255570	
SWNTTIDNI	9	H-2_Kd	356	89255570	
EHPSSSII	8	H-2_Dd	227	89255570	
MAYIIGIVFL	10	H-2_Kb	97	89255570	
LTPYQAIAL	9	H-2_Kb	53	89255591	L
IAFFLALII	9	H-2_Kb	476	89255591	
VFVTSAIYTVI	11	H-2_Kd	361	89255591	
VAALFLVWIIM	11	H-2_Kb	208	89255715	
ASLAKLLFCL	10	H-2_Kb	338	89255715	
FAIITSFL	8	H-2_Db	70	89255715	
LTLVITLLPPL	11	H-2_Kb	175	89255715	
VSVLLIVQPAM	11	H-2_Kb	52	89255715	
ILFSGLGIIAL	11	H-2_Kb	190	89255715	
LGIIALQLL	9	H-2_Kb	427	89255715	
FIPINVPM	8	H-2_Db	306	89255729	
IYIQSEAL	8	H-2_Kd	35	89255729	
KYLYPLGI	8	H-2_Kd	254	89255729	
KAYFMLMRL	9	H-2_Kb	30	89255776	
ISYLVIFTI	9	H-2_Kb	78	89255776	
VLFLNLYTI	9	H-2_Kb	455	89255776	
IGLIIFIIAV	10	H-2_Kb	283	89255776	
IGLIIFIIAVI	11	H-2_Kb	464	89255776	

LAPLILVGLML	11	H-2_Kb	405	89255853
SSYAYWLFL	9	H-2_Kb	39	89255853
LYLYLLAFCI	10	H-2_Kd	133	89255996
LYLYLLAFCII	11	H-2_Kd	72	89255996
LYLLAFCI	8	H-2_Kd	88	89255996
AILNIIVYL	9	H-2_Kb	132	89255996
IIFFVTFL	8	H-2_Kb	241	89255996
NIFISRTSL	9	H-2_Kb	434	89255996
YIYIKKEKL	9	H-2_Kb	333	89255996
LALFFLSIWIL	11	H-2_Kb	137	89255996
SNYITQLYTSL	11	H-2_Kb	22	89256001
YYFVYQTI	8	H-2_Kb	182	89256001
IAIQPRMTMLL	11	H-2_Kb	89	89256001
YNYGVYTTALL	11	H-2_Kb	237	89256001
ITIVCFLLSL	10	H-2_Kb	206	89256001
TSFGYRLLL	9	H-2_Kb	123	89256054
CIFLYSFL	8	H-2_Kb	37	89256054
YSISLIYTSI	10	H-2_Db	245	89256054
VFYALFIIL	9	H-2_Kb	76	89256054
LAIALFGHL	9	H-2_Kb	252	89256054
FLLRTYAWVTL	11	H-2_Db	461	89256070
IVILVLPIL	9	H-2_Kb	211	89256070
SVLGYYHRL	9	H-2_Kb	53	89256087
YAYIAFLIL	9	H-2_Db	318	89256124
IVIYSPQDM	9	H-2_Kb	379	89256124
IAFYKNIINI	10	H-2_Kb	135	89256124
FYNIIFSFI	9	H-2_Kd	7	89256124
SYLQPTEGIQI	11	H-2_Kd	220	89256124
VGWIYDAQKL	10	H-2_Kb	264	89256124
GYIFLPMPII	10	H-2_Kd	194	89256124
KTIKLYENL	9	H-2_Kb	250	89256142
IYIIILQYL	9	H-2_Kb	315	89256142
ISFNFRTI	8	H-2_Kb	11	89256223
GYISLLKMLII	11	H-2_Kd	154	89256223
VDITFSTV	8	H-2_Kb	88	89256223
VVLISLICM	9	H-2_Kb	365	89256223
VLISLICML	9	H-2_Kb	361	89256223
FGLPWQLIAL	10	H-2_Kb	356	89256223
AIIDSFRTPL	10	H-2_Kb	223	89256223
MKYIYKKL	8	H-2_Kb	17	89256227
SYAELNNQISI	11	H-2_Kd	22	89256227
NIYLSTARL	9	H-2_Kb	253	89256227
GGPSTSGIA	9	H-2_Dd	198	89256227
VGFNTGYVYDL	11	H-2_Kb	257	89256227
FYNRNDHLFVI	11	H-2_Kd	80	89256270
KSIIHGLQFI	10	H-2_Db	291	89256270
LAIVIVNLL	9	H-2_Kb	329	89256270

YGFIGVYSSAL	11	H-2_Kb	229	89256270
NMYHFKILYLL	11	H-2_Kb	155	89256270
LYLLIIIFL	9	H-2_Kd	423	89256270
KYIYFSLAL	9	H-2_Kd	226	89256270
LSFLRYEDVV	10	H-2_Kb	433	89256270
SAISTFLFL	9	H-2_Db	41	89256270
SNIILIFATIL	11	H-2_Kb	416	89256270
IILIFATIL	9	H-2_Kb	144	89256270
ITLFKKASRL	10	H-2_Kb	70	89256312
TLFKKASRL	9	H-2_Kb	331	89256312
AVLYSTLVL	9	H-2_Kb	425	89256312
KQSYNTLFL	9	H-2_Db	391	89256312
QYPLNTNYI	9	H-2_Db	174	89256312
NYIALVVGIL	10	H-2_Kd	442	89256312
FQNVNTVVIAV	11	H-2_Db	405	89256312
QLPSGNILA	9	H-2_Dd	105	89256315
FAFLGAFLLSM	11	H-2_Kb	132	89256315
LAILLFLTVM	10	H-2_Kb	112	89256315
FMGLSIAFL	9	H-2_Db	67	89256352
GYITLNISLII	11	H-2_Kd	66	89256377
SFLTIQQL	8	H-2_Kd	178	89256377
FYYKSSFIKVI	11	H-2_Kd	178	89256377
TLGFLFVAL	9	H-2_Kb	346	89256404
LVYLPLEAQSV	11	H-2_Kb	452	89256404
VGLIMYISL	9	H-2_Kb	8	89256404
FSLHIIYTI	9	H-2_Db	317	89256404
SSLVLFSCL	9	H-2_Kb	149	89256404
SMVMLIIFI	9	H-2_Db	55	89256517
SLFIKFHPI	9	H-2_Kb	329	89256517
VVYIGIFPGAL	11	H-2_Kb	33	89256517
LGIPVFFYYIL	11	H-2_Kb	245	89256552
PVFFYYILGLM	11	H-2_Kb	262	89256552
VAFRFPEFI	9	H-2_Kb	179	89256614
QFFKTLAAI	9	H-2_Kd	100	89256614
VDFTYLAM	8	H-2_Kb	87	89256614
GYLMSLFCFVV	11	H-2_Kd	497	89256614
LYAQNKTTIV	10	H-2_Kd	93	89256614
FYISLVSLI	9	H-2_Kd	4	89256614
SGDKYAFV	8	H-2_Kb	281	89256614
TYLPFMTIV	9	H-2_Kd	9	89256614
STIVEWYDFM	10	H-2_Db	80	89256710
FMLFAYLTPV	10	H-2_Db	73	89256710
IILVVMRL	8	H-2_Kb	116	89256710
SFATMSSGTGV	11	H-2_Kd	142	89256710
LALLTFIAL	9	H-2_Db	95	89256785
IGFVIPLV	8	H-2_Kb	498	89256785
IGFVIPLVSSL	11	H-2_Kb	69	89256785

ISFISGFGSAL	11	H-2_Kb	75	89256785	
GYISGAFIAL	10	H-2_Kd	16	89256785	
LIFLAIMAM	9	H-2_Kb	125	89256785	
LAIMAMFESI	10	H-2_Kb	419	89256785	
IMAMFESIMPL	11	H-2_Kb	94	89256785	
SYILQYILL	9	H-2_Db	458	89256788	
AMYVLVGVTM	10	H-2_Kb	110	89256788	
IVLPYAMI	8	H-2_Kb	92	89256788	
KILKNAGPYCI	11	H-2_Db	301	89256788	
LGFFIIQYL	9	H-2_Kb	31	89256851	
QYLNSQDKGL	10	H-2_Kd	102	89256851	
IIMLLICSIL	10	H-2_Kb	381	89256851	
HYFKGLEV	8	H-2_Kd	407	89256892	
LAIVLFYLL	9	H-2_Kb	266	89256892	
FVVLAFFII	9	H-2_Db	322	89256892	
ANYLLFNYL	9	H-2_Kb	10	89256892	
FNYLGNPGL	9	H-2_Kb	280	89256892	
VVIRFWIISL	10	H-2_Kb	34	89256892	
KLVVSLIIFI	10	H-2_Db	491	89256896	
HFAPIKYAI	9	H-2_Kd	437	89256896	
QIYFEKNKTL	10	H-2_Kb	301	89256896	
IYFEKNKTL	9	H-2_Kb	410	89256896	
LYPLIFFFIL	10	H-2_Kb	474	89256896	
DSPSYNQMSL	10	H-2_Dd	103	89256896	
LAAIMIMMVGL	11	H-2_Kb	280	89256917	
IGLGYIAV	8	H-2_Kb	120	89256917	
IGYMAAPI	8	H-2_Kb	52	89256917	
IGYMAAPISM	10	H-2_Kb	14	89256917	
TYFEGIIGAI	10	H-2_Kd	37	89256917	
FSATNYYEVA	10	H-2_Db	294	89256946	L
YSYDFFIRAAP	11	H-2_Kb	157	89256946	
VAYIGRIFMGI	11	H-2_Kb	139	89256946	
LFIPINVLGSL	11	H-2_Kd	57	89256946	
VGLIQAKFGL	10	H-2_Kb	229	89256946	
TSYQNALDMVI	11	H-2_Db	178	89256946	
SYQNALDMVI	10	H-2_Kd	37	89256946	
LSFTNAVFESV	11	H-2_Kb	416	89256977	
VYLLLTFLCFI	11	H-2_Kd	200	89256977	
HYIALSKFKI	10	H-2_Kd	94	89256977	
FYFLFFTSFI	10	H-2_Kd	10	89256977	
FYFLFFTSFIV	11	H-2_Kd	376	89256977	
IAHTNDLSNI	10	H-2_Db	176	89256977	
SIVTNSIFQVI	11	H-2_Db	185	89256977	
TTLSNVGPGL	10	H-2_Kb	214	89256977	
FALLLYMIL	9	H-2_Db	28	89257033	
QGYIFGEL	8	H-2_Kb	31	89257033	
TGITLIFGYSL	11	H-2_Kb	221	89257033	

YSLLGATRLIL	11	H-2_Db	75	89257033
LAVLMLVIGGM	11	H-2_Kb	414	89257033
SMLTLIFPYI	10	H-2_Kb	412	89257033
LIPAIIMIPLL	11	H-2_Kb	281	89257033
CQLIIIAAM	9	H-2_Kb	83	89257093
VNAWNVLAYTM	11	H-2_Kb	500	89257093
TMILRAALAL	10	H-2_Kb	142	89257093
TAIGIAIGPL	10	H-2_Kb	375	89257093
SYNQIFLICAV	11	H-2_Kd	58	89257093
SLLLFSAPI	9	H-2_Kb	498	89257093
IILAFYHPM	9	H-2_Kb	12	89257107
FYHPMFLV	8	H-2_Kd	187	89257107
HSYKYGDILL	10	H-2_Kb	248	89257164
IIAFIYSLL	9	H-2_Kb	187	89257164
FIYSLLAGFSL	11	H-2_Kb	121	89257164
IVLQQYKSKL	10	H-2_Kb	48	89257164
LSFIGINLWLL	11	H-2_Kb	128	89257164
TSTIITFFISL	11	H-2_Kb	149	89257191
IITFFISL	8	H-2_Kb	61	89257191
