# Supplementary material for: Clusters versus Affinity-Based Approaches in F. tularensis Whole Genome Search of CTL Epitopes
Source: PLoS One. 2012 May 1;7(5):e36440. doi: 10.1371/journal.pone.0036440 (PMC3341354; doi:10.1371/journal.pone.0036440)
Supplement: Table S3 — List of 92 peptides selected from the high affinity-based approach, having IC50 values< = 10 nM. The affinity provided is the IC50 value predicted for a particular responder sequence by the NetMHC3.0 program. The gi number and annotation of the source protein are according to the F. tularensis holarctica LVS sequence deposited at the NCBI (GenBank accession AM233362); (a) Responders are indicated by their magnitude of T-cell response as follows number of spots/million cells) is: L (Low): 5–20; M (medium) - 20–32; H (high) - 33 and above. (PDF) [file pone.0036440.s003.pdf]

## Table S3: Compilation of data for high affinity-based selected putative MHC binders ($IC_{50}$ < = 10 nM, Subset V)

| Sequence | Length | Predicted allele | Affinity ($IC_{50}$, nM) | Protein gi# | Responder[(a)] |
|---|---|---|---|---|---|
| KAYKNSYFL | 9 | H-2_Db | 4 | 89255452 | L |
| FSIPNSDKI | 9 | H-2_Db | 10 | 89255460 | |
| CSIENIDPM | 9 | H-2_Db | 5 | 89255476 | |
| VSYWFSPYL | 9 | H-2_Kb | 5 | 89255497 | |
| LGYTYQRV | 8 | H-2_Kb | 6 | 89255508 | |
| SSRLNKIPL | 9 | H-2_Db | 10 | 89255530 | |
| ISLNNFVSL | 9 | H-2_Kb | 9 | 89255554 | |
| IAFTKYPSL | 9 | H-2_Kb | 7 | 89255563 | |
| FGLRNGFVI | 9 | H-2_Db | 9 | 89255584 | |
| FSPQYALL | 8 | H-2_Kb | 8 | 89255588 | |
| HSYTFTGV | 8 | H-2_Kb | 10 | 89255612 | |
| YSIGFYNM | 8 | H-2_Kb | 10 | 89255620 | |
| YGYPYAYL | 8 | H-2_Kb | 3 | 89255698 | M |
| YQYVNPYIL | 9 | H-2_Db | 7 | 89255720 | M |
| LSIKNAIWL | 9 | H-2_Db | 10 | 89255776 | |
| ISFIFLFAIAM | 11 | H-2_Kb | 10 | 89255792 | |
| AAFTNLLAM | 9 | H-2_Db | 9 | 89255796 | |
| YHYGYYRL | 8 | H-2_Kb | 10 | 89255799 | |
| FSLGNAFVI | 9 | H-2_Db | 6 | 89255849 | |
| LMILNLLPI | 9 | H-2_Db | 4 | 89255858 | |
| TNYFNYILPPL | 11 | H-2_Kb | 8 | 89255886 | H |
| ISYAKFAANTL | 11 | H-2_Kb | 7 | 89255905 | M |
| QSLLNQYSM | 9 | H-2_Db | 6 | 89255942 | |
| VSWIFYLL | 8 | H-2_Kb | 6 | 89255968 | |
| IIIVYKFL | 8 | H-2_Kb | 10 | 89255979 | |
| VTLTNQDII | 9 | H-2_Db | 10 | 89256007 | |
| ISFIYGSI | 8 | H-2_Kb | 7 | 89256019 | |
| IVYWFGSL | 8 | H-2_Kb | 4 | 89256021 | |
| LGYYYYVMIL | 10 | H-2_Kb | 9 | 89256057 | |
| INYFYYEV | 8 | H-2_Kb | 4 | 89256059 | |
| ASPSNFIAM | 9 | H-2_Db | 10 | 89256078 | |
| AQYQFLNPL | 9 | H-2_Kb | 4 | 89256080 | |
| NMVSNTYTL | 9 | H-2_Db | 6 | 89256149 | |
| FTFKNHLFM | 9 | H-2_Db | 10 | 89256161 | |
| SILIFAYL | 8 | H-2_Kb | 10 | 89256199 | M |
| SAYLNFVNTL | 10 | H-2_Kb | 6 | 89256211 | |
| KALKNPVEI | 9 | H-2_Db | 6 | 89256235 | |
| LSLLFSPL | 8 | H-2_Kb | 7 | 89256294 | |
| ISFEYPLI | 8 | H-2_Kb | 8 | 89256308 | |
| FGFTFQSL | 8 | H-2_Kb | 8 | 89256308 | |
| LSYVFQSI | 8 | H-2_Kb | 4 | 89256312 | |
| YAVKNNFFYM | 10 | H-2_Db | 3 | 89256315 | |

| | | | | | |
|---|---|---|---|---|---|
| SAYGYAQV | 8 | H-2_Kb | 6 | 89256336 | |
| ISYIKNNPL | 9 | H-2_Kb | 8 | 89256389 | |
| SSYFLYALL | 9 | H-2_Kb | 7 | 89256418 | |
| TIYLFEGL | 8 | H-2_Kb | 6 | 89256424 | |
| VSYAYFNL | 8 | H-2_Kb | 2 | 89256432 | |
| RIYGYSKL | 8 | H-2_Kb | 5 | 89256519 | |
| IMLINNIFM | 9 | H-2_Db | 5 | 89256526 | |
| FAIFNGAFL | 9 | H-2_Db | 5 | 89256526 | |
| IAYMRSQFDSL | 11 | H-2_Kb | 7 | 89256533 | |
| FALVNRGEI | 9 | H-2_Db | 4 | 89256534 | |
| FSILYINI | 8 | H-2_Kb | 10 | 89256534 | |
| SNIKYNQL | 8 | H-2_Kb | 6 | 89256540 | |
| FNLLNKDAL | 9 | H-2_Db | 8 | 89256547 | |
| STYFRINAM | 9 | H-2_Kb | 7 | 89256549 | |
| MGYKYQEL | 8 | H-2_Kb | 3 | 89256588 | |
| TVLYFIPYL | 9 | H-2_Kb | 9 | 89256604 | |
| IIYLYGDL | 8 | H-2_Kb | 7 | 89256616 | |
| TSYEFELL | 8 | H-2_Kb | 5 | 89256631 | L |
| YAYNYANV | 8 | H-2_Kb | 5 | 89256637 | |
| IALYTLISL | 9 | H-2_Kb | 10 | 89256644 | M |
| IIYAFTPC | 8 | H-2_Kb | 9 | 89256655 | |
| TSLQNEQYM | 9 | H-2_Db | 7 | 89256673 | |
| VANQNVIVI | 9 | H-2_Db | 10 | 89256776 | |
| YALINNASTI | 10 | H-2_Db | 7 | 89256795 | |
| IVLEYSSL | 8 | H-2_Kb | 8 | 89256824 | |
| IAIRYGNL | 8 | H-2_Kb | 4 | 89256845 | L |
| YALMNYII | 8 | H-2_Db | 10 | 89256859 | |
| LSLLNIIFI | 9 | H-2_Db | 5 | 89256899 | |
| SSIIYLNV | 8 | H-2_Kb | 10 | 89256901 | |
| ASLMFSPL | 8 | H-2_Kb | 9 | 89256905 | |
| IGYYWKPGL | 9 | H-2_Kb | 8 | 89256912 | |
| VIYSFLTL | 8 | H-2_Kb | 8 | 89256912 | |
| YSFIFFISL | 9 | H-2_Kb | 8 | 89256943 | |
| ASLTNRYRL | 9 | H-2_Db | 8 | 89256946 | |
| TAYQFTQPI | 9 | H-2_Kb | 10 | 89256986 | |
| ISVIYACL | 8 | H-2_Kb | 5 | 89256994 | |
| AMSLNITYI | 9 | H-2_Db | 10 | 89257000 | |
| KSIVYSLL | 8 | H-2_Kb | 6 | 89257022 | |
| IGFLYTKL | 8 | H-2_Kb | 4 | 89257022 | |
| MTPLYVKL | 8 | H-2_Kb | 9 | 89257033 | |
| FMMLTVVYL | 9 | H-2_Db | 8 | 89257064 | |
| SSYQYSYV | 8 | H-2_Kb | 4 | 89257112 | |
| MGFSYTQV | 8 | H-2_Kb | 8 | 89257125 | |
| AAYRYIQTM | 9 | H-2_Kb | 10 | 89257128 | |
| FQYTNLTIL | 9 | H-2_Db | 7 | 89257130 | |
| FNLLNRIKL | 9 | H-2_Db | 10 | 89257153 | |
| SSYEFTQL | 8 | H-2_Kb | 2 | 89257154 | |
| TMPVNYDAI | 9 | H-2_Db | 5 | 89257177 | |
| FAVSNFFII | 9 | H-2_Db | 5 | 89257184 | |
| FSLLNPQAI | 9 | H-2_Db | 5 | 89257190 | |
