# Supplementary material for: Clusters versus Affinity-Based Approaches in F. tularensis Whole Genome Search of CTL Epitopes
Source: PLoS One. 2012 May 1;7(5):e36440. doi: 10.1371/journal.pone.0036440 (PMC3341354; doi:10.1371/journal.pone.0036440)
Supplement: Table S4 — List of 370 peptides (262 selected by the cluster-based approach - indicated as “clu" and 108 selected by the affinity-based approach - indicated as “aff") tested in the BALB/c screen. The affinity provided is the IC50 value predicted for a particular responder sequence by the NetMHC3.0 program. The gi number and annotation of the source protein are according to the F. tularensis holarctica LVS sequence deposited at the NCBI (GenBank accession AM233362); (a) Responders are indicated by their magnitude of T-cell response as follows (number of spots/million cells) : L (Low): 5–20; M (medium) - 20–32; H (high) - 33 and above. (PDF) [file pone.0036440.s004.pdf]

## Table S4: Compilation of predicted MHC binders evaluted in LVS-immunized mice

| Sequence | Length | Predicted allele | Affinity ($IC_{50}$, nM) | Protein gi# | Responder[a] |
|---|---|---|---|---|---|
| LYLFAILPI | 9 | Kd | 299 | 89255471 | L |
| LFLPIQTGI | 9 | Kd | 8 | 89255471 | |
| VYLSKNGLTSI | 11 | Kd | 9 | 89255473 | |
| SYLVLLANSL | 10 | Kd | 7 | 89255490 | |
| FYYALINI | 8 | Kd | 110 | 89255508 | |
| FYYALINIL | 9 | Kd | 72 | 89255508 | |
| FYYALINILL | 10 | Kd | 253 | 89255508 | |
| YYALINIL | 8 | Kd | 266 | 89255508 | |
| HYIFASVL | 8 | Kd | 36 | 89255524 | |
| HYIFASVLL | 9 | Kd | 347 | 89255524 | |
| HYIFASVLLVL | 11 | Kd | 208 | 89255524 | |
| SYHYIFASV | 9 | Kd | 90 | 89255524 | |
| SYHYIFASVL | 10 | Kd | 42 | 89255524 | |
| SYHYIFASVLL | 11 | Kd | 370 | 89255524 | |
| KYLNSNLSAL | 10 | Kd | 5 | 89255538 | |
| LYLSFSEGSI | 10 | Kd | 10 | 89255552 | |
| CYLPGFLSSVI | 11 | Kd | 9 | 89255553 | |
| SYQGKWSI | 8 | Kd | 10 | 89255562 | |
| SYIINYKL | 8 | Kd | 297 | 89255563 | |
| SYIINYKLLNI | 11 | Kd | 421 | 89255563 | M |
| KYLGVPIFL | 9 | Kd | 24 | 89255570 | L |
| KYLGVPIFLL | 10 | Kd | 139 | 89255570 | |
| KYLGVPIFLLM | 11 | Kd | 506 | 89255570 | |
| MYLMFLFSI | 9 | Kd | 89 | 89255570 | |
| LYISFTGTQI | 10 | Kd | 5 | 89255571 | |
| AYVQVTSPL | 9 | Kd | 7 | 89255575 | |
| HYVGMQHSV | 9 | Kd | 6 | 89255576 | |
| SYAKINLFL | 9 | Kd | 110 | 89255588 | |
| SYAKINLFLHI | 11 | Kd | 72 | 89255588 | |
| SSSYQNVSY | 9 | Dd | 785 | 89255591 | |
| SYMTVVNKV | 9 | Kd | 34 | 89255591 | |
| SYMTVVNKVL | 10 | Kd | 20 | 89255591 | |
| SYMTVVNKVLI | 11 | Kd | 11 | 89255591 | |
| SYQNVSYM | 8 | Kd | 116 | 89255591 | L |
| SYQNVSYMTV | 10 | Kd | 175 | 89255591 | |
| SYQNVSYMTVV | 11 | Kd | 62 | 89255591 | |
| LYIRLTAL | 8 | Kd | 5 | 89255592 | |
| SYIKLESIV | 9 | Kd | 5 | 89255620 | |
| GYTTMVWASI | 10 | Kd | 9 | 89255626 | |

| | | | | | |
|---|---|---|---|---|---|
| LYVNNTTTI | 9 | Kd | 7 | 89255631 | |
| LYQNRYTAI | 9 | Kd | 8 | 89255635 | |
| KYIATITSL | 9 | Kd | 1 | 89255651 | |
| TYKTTITSMI | 10 | Kd | 9 | 89255654 | |
| FYLLSQSDHYI | 11 | Kd | 5 | 89255698 | |
| MYLHFNYI | 8 | Kd | 207 | 89255714 | |
| NYILIIAL | 8 | Kd | 298 | 89255714 | |
| SFMYLHFNYI | 10 | Kd | 928 | 89255714 | |
| TFCAISTI | 8 | Kd | 201 | 89255714 | |
| AYIFMGGQL | 9 | Kd | 14 | 89255715 | |
| FYLLLLYSL | 9 | Kd | 15 | 89255715 | |
| FYLLLLYSLL | 10 | Kd | 21 | 89255715 | |
| LYSLLTAYI | 9 | Kd | 498 | 89255715 | |
| AYISAAASSL | 10 | Kd | 6 | 89255723 | |
| TYVAIMTSFL | 10 | Kd | 5 | 89255723 | |
| LYIFKIGL | 8 | Kd | 269 | 89255729 | |
| LYIFKIGLL | 9 | Kd | 632 | 89255729 | |
| SFVIMVMLTGL | 11 | Kd | 135 | 89255729 | |
| IYKSIASQTEI | 11 | Kd | 10 | 89255737 | |
| FYQGHLSPI | 9 | Kd | 10 | 89255740 | |
| FYLLGKDKL | 9 | Kd | 9 | 89255745 | |
| TYNTDKTQTDI | 11 | Kd | 3 | 89255754 | |
| LYLLAVAL | 8 | Kd | 78 | 89255755 | |
| LYLLAVALRL | 10 | Kd | 952 | 89255755 | |
| LYILSFVSSL | 10 | Kd | 6 | 89255759 | |
| FYLDRRTEI | 9 | Kd | 6 | 89255762 | |
| TFLTTLLAPI | 10 | Kd | 101 | 89255770 | |
| LFLNLYTIL | 9 | Kd | 368 | 89255776 | |
| LYTILLSFV | 9 | Kd | 250 | 89255776 | H |
| LYTILLSFVAL | 11 | Kd | 135 | 89255776 | |
| RYILLFNFL | 9 | Kd | 17 | 89255776 | |
| RYILLFNFLSL | 11 | Kd | 48 | 89255776 | |
| GYLFGNTI | 8 | Kd | 12 | 89255790 | |
| GYLFGNTIL | 9 | Kd | 6 | 89255790 | |
| KYLKDTTESKI | 11 | Kd | 10 | 89255800 | M |
| FYIIITGI | 8 | Kd | 7 | 89255814 | M |
| KYCLLNEPI | 9 | Kd | 6 | 89255818 | M |
| SYMRTDSTNL | 10 | Kd | 5 | 89255836 | |
| FFLALLNYV | 9 | Kd | 189 | 89255853 | |
| FYSILLTRI | 9 | Kd | 14 | 89255853 | L |
| FYSILLTRIL | 10 | Kd | 726 | 89255853 | |
| FYSILLTRILL | 11 | Kd | 284 | 89255853 | |
| GFYSILLTRI | 10 | Kd | 659 | 89255853 | |
| KYYYNTLI | 8 | Kd | 90 | 89255853 | |
| KYYYNTLI | 8 | Kd | 90 | 89255853 | |
| YYNTLIIL | 8 | Kd | 86 | 89255853 | |
| YYNTLIIL | 8 | Kd | 86 | 89255853 | |

| | | | | | |
|---|---|---|---|---|---|
| YYNTLIILL | 9 | Kd | 214 | 89255853 | |
| YYNTLIILL | 9 | Kd | 214 | 89255853 | |
| YYNTLIILLL | 10 | Kd | 479 | 89255853 | |
| YYNTLIILLL | 10 | Kd | 479 | 89255853 | |
| YYYNTLII | 8 | Kd | 696 | 89255853 | |
| YYYNTLII | 8 | Kd | 696 | 89255853 | |
| KYNHASSMTTI | 11 | Kd | 5 | 89255875 | |
| LYRQMHSPI | 9 | Kd | 4 | 89255875 | |
| KYNLIAVATNL | 11 | Kd | 8 | 89255882 | |
| SYGTGSNHI | 9 | Kd | 5 | 89255894 | |
| SYINGTDGI | 9 | Kd | 10 | 89255940 | M |
| WYVFMATKL | 9 | Kd | 6 | 89255940 | |
| KYNINTSTSL | 10 | Kd | 5 | 89255955 | |
| FYLLMFLIAV | 10 | Kd | 80 | 89255968 | |
| NYIVLTSV | 8 | Kd | 33 | 89255968 | |
| NYIVLTSVSWI | 11 | Kd | 84 | 89255968 | |
| NYIQLLSYL | 9 | Kd | 8 | 89255971 | |
| GYAPVLYFM | 9 | Kd | 755 | 89255979 | |
| LYFMGACI | 8 | Kd | 57 | 89255979 | |
| LYFMGACIV | 9 | Kd | 285 | 89255979 | |
| LYFMGACIVV | 10 | Kd | 508 | 89255979 | |
| NYGYAPVLYF | 10 | Dd | 831 | 89255979 | |
| TYNYGYAPV | 9 | Kd | 191 | 89255979 | |
| TYNYGYAPVL | 10 | Kd | 450 | 89255979 | |
| YYLNGYSANSI | 11 | Kd | 4 | 89255987 | |
| FFFFFLSMI | 9 | Kd | 712 | 89255996 | |
| FFLSMIYL | 8 | Kd | 249 | 89255996 | |
| FFLSMIYLI | 9 | Kd | 814 | 89255996 | |
| FYILIIFL | 8 | Kd | 58 | 89255996 | |
| GYIKYLYYYGI | 11 | Kd | 547 | 89255996 | |
| IYLIIVQI | 8 | Kd | 279 | 89255996 | |
| KYLYYYGI | 8 | Kd | 430 | 89255996 | |
| KYLYYYGII | 9 | Kd | 303 | 89255996 | L |
| LYFEMRKCFI | 10 | Kd | 21 | 89255996 | |
| CYVSTNIM | 8 | Kd | 146 | 89256001 | |
| LFICYVSTNI | 10 | Kd | 822 | 89256001 | |
| LYLVCIAI | 8 | Kd | 99 | 89256001 | |
| MYYFVYQTII | 10 | Kd | 132 | 89256001 | |
| VYQTIIAI | 8 | Kd | 98 | 89256001 | |
| VYQTIIAIL | 9 | Kd | 409 | 89256001 | |
| VYQTIIAILYL | 11 | Kd | 555 | 89256001 | |
| YYFVYQTI | 8 | Kd | 82 | 89256001 | |
| YYFVYQTII | 9 | Kd | 23 | 89256001 | |
| YYFVYQTIIAI | 11 | Kd | 85 | 89256001 | |
| SYIGGVVAL | 9 | Kd | 10 | 89256019 | |
| FFSSMLSLV | 9 | Kd | 410 | 89256021 | |
| KYYFVLLLAFL | 11 | Kd | 167 | 89256054 | |

| | | | | | |
|---|---|---|---|---|---|
| YYFVLLLAFL | 10 | Kd | 165 | 89256054 | |
| YYVMILAV | 8 | Kd | 258 | 89256057 | |
| YYVMILAVAI | 10 | Kd | 25 | 89256057 | L |
| YYYVMILAV | 9 | Kd | 701 | 89256057 | |
| YYYVMILAVAI | 11 | Kd | 98 | 89256057 | |
| YYYYVMILAV | 10 | Kd | 967 | 89256057 | |
| KYNNSEKSPL | 10 | Kd | 6 | 89256059 | |
| HYNLIFISL | 9 | Kd | 34 | 89256070 | |
| HYNLIFISLL | 10 | Kd | 75 | 89256070 | |
| LYLSNYIELI | 10 | Kd | 249 | 89256070 | |
| NYIELIHYNL | 10 | Kd | 941 | 89256070 | |
| NYIELIHYNLI | 11 | Kd | 783 | 89256070 | |
| LYNLSNSSI | 9 | Kd | 3 | 89256084 | |
| VYMKINSQI | 9 | Kd | 6 | 89256084 | |
| KYFFVLLI | 8 | Kd | 478 | 89256087 | |
| KYFFVLLIL | 9 | Kd | 553 | 89256087 | |
| KYFFVLLILFI | 11 | Kd | 303 | 89256087 | |
| FYFSIISYYI | 10 | Kd | 23 | 89256094 | M |
| IYNYSSKI | 8 | Kd | 475 | 89256094 | |
| YYIYNYSSKI | 10 | Kd | 67 | 89256094 | |
| KFVTLGTISAI | 11 | Kd | 8 | 89256111 | |
| AYIAFLIL | 8 | Kd | 847 | 89256124 | |
| CYPMNIAALYF | 11 | Dd | 757 | 89256124 | |
| FYAYIAFL | 8 | Kd | 391 | 89256124 | |
| FYAYIAFLI | 9 | Kd | 855 | 89256124 | |
| FYNIIFSFI | 9 | Kd | 7 | 89256124 | M |
| LYHNFKTKI | 9 | Kd | 9 | 89256140 | |
| IYIIILQYL | 9 | Kd | 105 | 89256142 | |
| IYIIILQYLL | 10 | Kd | 575 | 89256142 | |
| QYLLSPIV | 8 | Kd | 823 | 89256142 | |
| SYLPAYSNL | 9 | Kd | 4 | 89256148 | |
| FYNKDKGYGFI | 11 | Kd | 9 | 89256155 | |
| KYSILLYSL | 9 | Kd | 233 | 89256177 | |
| LYSLTTLL | 8 | Kd | 298 | 89256177 | |
| FYCGGGFRSAL | 11 | Kd | 7 | 89256194 | |
| FKGYNYGNYTL | 11 | Dd | 931 | 89256197 | |
| GYNYGNYTL | 9 | Dd | 159 | 89256197 | |
| GYNYGNYTLL | 10 | Dd | 462 | 89256197 | |
| NYTLLLSGV | 9 | Kd | 761 | 89256197 | |
| NYTLLLSGVI | 10 | Kd | 542 | 89256197 | |
| GYEKAFTTI | 9 | Kd | 4 | 89256208 | |
| KYSHIQTII | 9 | Kd | 8 | 89256218 | |
| CYIALIIV | 8 | Kd | 759 | 89256223 | |
| CYIALIIVL | 9 | Kd | 260 | 89256223 | |
| CYIALIIVLI | 10 | Kd | 182 | 89256223 | |
| LYFILTCYI | 9 | Kd | 76 | 89256223 | |
| YFILTCYI | 8 | Kd | 327 | 89256223 | |

| | | | | | |
|---|---|---|---|---|---|
| VYYLVLNMHI | 10 | Kd | 200 | 89256227 | |
| YYLVLNMHI | 9 | Kd | 29 | 89256227 | |
| SYQLFSSL | 8 | Kd | 10 | 89256263 | |
| FYGVLLIFFI | 10 | Kd | 295 | 89256270 | L |
| FYLLVNLNV | 9 | Kd | 307 | 89256270 | |
| FYLLVNLNVF | 10 | Kd | 819 | 89256270 | |
| AYLVAPSL | 8 | Kd | 80 | 89256276 | |
| AYLVAPSLI | 9 | Kd | 41 | 89256276 | |
| FYISINLGSL | 10 | Kd | 5 | 89256276 | |
| FYISINLGSLL | 11 | Kd | 10 | 89256276 | |
| FYLLTKDNI | 9 | Kd | 4 | 89256308 | M |
| LFIALQYPL | 9 | Kd | 592 | 89256312 | |
| SYLTLFTGLTI | 11 | Kd | 10 | 89256312 | |
| QYIGIFLAI | 9 | Kd | 18 | 89256315 | |
| QYIGIFLAIL | 10 | Kd | 59 | 89256315 | |
| QYIGIFLAILL | 11 | Kd | 116 | 89256315 | |
| SYNTLQDI | 8 | Kd | 7 | 89256320 | |
| SYLAALIAI | 9 | Kd | 15 | 89256352 | |
| SYLAALIAII | 10 | Kd | 49 | 89256352 | |
| SYLAALIAIII | 11 | Kd | 81 | 89256352 | L |
| KYITMVNDL | 9 | Kd | 2 | 89256376 | |
| IYFTSLSNQI | 10 | Kd | 8 | 89256377 | |
| IYFTSLSNQIL | 11 | Kd | 41 | 89256377 | |
| LYLFLVIVMI | 10 | Kd | 383 | 89256377 | |
| YFTSLSNQI | 9 | Kd | 759 | 89256377 | |
| MYLNLDTEI | 9 | Kd | 6 | 89256392 | |
| AFIIMLSLTYL | 11 | Kd | 67 | 89256404 | |
| SYYGVFVAF | 9 | Kd | 758 | 89256404 | |
| SYYGVFVAFI | 10 | Kd | 79 | 89256404 | |
| SYYGVFVAFII | 11 | Kd | 137 | 89256404 | |
| TYLNTYKL | 8 | Kd | 512 | 89256404 | |
| KYIRGYFSL | 9 | Kd | 4 | 89256410 | H |
| KYMGMTLL | 8 | Kd | 7 | 89256429 | |
| KYMGMTLLAL | 10 | Kd | 20 | 89256429 | |
| NYFKYMGMTLL | 11 | Kd | 144 | 89256429 | |
| KYMGMTLL | 8 | Kd | 7 | 89256429 | |
| HYQTIQEEL | 9 | Kd | 6 | 89256430 | |
| YYQFLIQL | 8 | Kd | 232 | 89256432 | |
| GFISALIYVYL | 11 | Kd | 747 | 89256438 | |
| VYIFGFISALI | 11 | Kd | 422 | 89256438 | |
| FYIISISCSI | 10 | Kd | 4 | 89256459 | |
| SYLYGLEHL | 9 | Kd | 8 | 89256475 | |
| IYLRMYVEASI | 11 | Kd | 7 | 89256498 | |
| GYLYGFSAL | 9 | Kd | 5 | 89256504 | |
| SYNKTIEYI | 9 | Kd | 5 | 89256509 | |
| YFMPIISL | 8 | Kd | 770 | 89256517 | |
| CYNRLKSGI | 9 | Kd | 3 | 89256523 | |

| | | | | | |
|---|---|---|---|---|---|
| HYNPVNNNL | 9 | Kd | 4 | 89256551 | |
| SYTTIKAFL | 9 | Kd | 8 | 89256551 | |
| AYYWLLTAL | 9 | Kd | 19 | 89256552 | |
| MYLMMFFAAL | 10 | Kd | 35 | 89256552 | |
| WLLTALSTQI | 10 | Kd | 878 | 89256552 | |
| YWLLTALSTQI | 11 | Kd | 488 | 89256552 | |
| YYWLLTAL | 8 | Kd | 13 | 89256552 | |
| SYSAAKSAL | 9 | Kd | 4 | 89256563 | |
| CYYTLRNNI | 9 | Kd | 8 | 89256576 | |
| FYNGAIETIL | 10 | Kd | 8 | 89256596 | |
| YYITKATWI | 9 | Kd | 7 | 89256603 | |
| LFLLIITFL | 9 | Kd | 182 | 89256614 | |
| FYQKGTSL | 8 | Kd | 3 | 89256631 | |
| RYIQTMAPSNI | 11 | Kd | 8 | 89256632 | |
| KYLLVCAAI | 9 | Kd | 4 | 89256638 | |
| KYLKITNQEI | 10 | Kd | 7 | 89256639 | |
| FYGETISTL | 9 | Kd | 10 | 89256655 | |
| TYIPYQDQTNI | 11 | Kd | 5 | 89256673 | |
| AYANIMYYL | 9 | Kd | 693 | 89256710 | |
| LSAYANIMY | 9 | Dd | 670 | 89256710 | |
| MYYLVLSYL | 9 | Kd | 27 | 89256710 | |
| SYLSNHFV | 8 | Kd | 46 | 89256710 | |
| SYLSNHFVEL | 10 | Kd | 43 | 89256710 | |
| VYLVLAMI | 8 | Kd | 361 | 89256710 | |
| YYLVLSYL | 8 | Kd | 25 | 89256710 | |
| KYLPQNFI | 8 | Kd | 10 | 89256715 | |
| KYYQSATATIL | 11 | Kd | 4 | 89256715 | |
| KYILPTSPNYL | 11 | Kd | 10 | 89256722 | |
| TYFIMLFTL | 9 | Kd | 61 | 89256727 | |
| TYFIMLFTLF | 10 | Kd | 977 | 89256727 | |
| YFIMLFTL | 8 | Kd | 461 | 89256727 | |
| TYAPLLDGSL | 10 | Kd | 9 | 89256753 | |
| LYLTVGTGV | 9 | Kd | 5 | 89256781 | |
| GYISGAFI | 8 | Kd | 17 | 89256785 | |
| GYISGAFIAL | 10 | Kd | 16 | 89256785 | |
| GYISGAFIALI | 11 | Kd | 55 | 89256785 | |
| QYILLCVI | 8 | Kd | 12 | 89256788 | L |
| QYILLCVIM | 9 | Kd | 186 | 89256788 | |
| SYILQYIL | 8 | Kd | 200 | 89256788 | |
| NYLKTKSDGFI | 11 | Kd | 9 | 89256792 | |
| AYSPVMTII | 9 | Kd | 7 | 89256799 | M |
| KYDKLNTII | 9 | Kd | 6 | 89256806 | |
| CYIGSKEAL | 9 | Kd | 3 | 89256809 | |
| NYNIVKTNL | 9 | Kd | 10 | 89256809 | |
| FYIFISVV | 8 | Kd | 56 | 89256811 | |
| FYIFISVVL | 9 | Kd | 81 | 89256811 | |
| FYIFISVVLL | 10 | Kd | 175 | 89256811 | |

| | | | | | |
|---|---|---|---|---|---|
| FYIFISVVLLL | 11 | Kd | 329 | 89256811 | |
| LYLYNFTNNPL | 11 | Kd | 10 | 89256841 | |
| QYANSLNTSL | 10 | Kd | 3 | 89256842 | |
| SYSMIIVI | 8 | Kd | 536 | 89256851 | |
| SYSMIIVITI | 10 | Kd | 542 | 89256851 | |
| SYSMIIVITIL | 11 | Kd | 111 | 89256851 | |
| FYISAKNNIGI | 11 | Kd | 7 | 89256857 | |
| SYISVRII | 8 | Kd | 27 | 89256892 | |
| SYISVRIIM | 9 | Kd | 39 | 89256892 | H |
| SYISVRIIMI | 10 | Kd | 22 | 89256892 | |
| SYYLLLNDIGV | 11 | Kd | 492 | 89256893 | |
| YYLLLNDI | 8 | Kd | 19 | 89256893 | |
| YYLLLNDIGV | 10 | Kd | 492 | 89256893 | |
| GYISIPLAFI | 10 | Kd | 32 | 89256896 | L |
| GYISIPLAFII | 11 | Kd | 55 | 89256896 | |
| GYITIILNL | 9 | Kd | 112 | 89256896 | |
| GYITIILNLL | 10 | Kd | 31 | 89256896 | L |
| LYAQLFNL | 8 | Kd | 360 | 89256896 | L |
| LYAQLFNLSSL | 11 | Kd | 10 | 89256896 | |
| FYQKSQQEI | 9 | Kd | 5 | 89256898 | |
| CYGLSLSSTSL | 11 | Kd | 7 | 89256901 | |
| KNPSRNVP | 8 | Dd | 932 | 89256917 | |
| KNPSRNVPL | 9 | Dd | 37 | 89256917 | |
| KNPSRNVPLA | 10 | Dd | 596 | 89256917 | |
| KNPSRNVPLAI | 11 | Dd | 298 | 89256917 | |
| LYMGLQYAFM | 10 | Kd | 631 | 89256917 | H |
| QYAFMQAV | 8 | Kd | 120 | 89256917 | |
| KYLKICDTI | 9 | Kd | 3 | 89256942 | |
| IYTITSYQNAL | 11 | Kd | 299 | 89256946 | |
| LYVPMSLSM | 9 | Kd | 79 | 89256946 | |
| LYVPMSLSMF | 10 | Kd | 338 | 89256946 | |
| SYQNALDMV | 9 | Kd | 87 | 89256946 | |
| SYQNALDMVI | 10 | Kd | 37 | 89256946 | |
| SYQNALDMVII | 11 | Kd | 99 | 89256946 | L |
| SYTLAKSQL | 9 | Kd | 6 | 89256963 | |
| SYNPDTTI | 8 | Kd | 7 | 89256969 | |
| MFLSLTML | 8 | Kd | 493 | 89256977 | |
| MFLSLTMLSPL | 11 | Kd | 201 | 89256977 | |
| FYIKRDGEI | 9 | Kd | 9 | 89256996 | |
| LYFANILI | 8 | Kd | 702 | 89256999 | |
| TYVLASLL | 8 | Kd | 113 | 89256999 | |
| SYITMDNFI | 9 | Kd | 2 | 89257028 | |
| TYISMLTL | 8 | Kd | 9 | 89257033 | |
| TYISMLTLI | 9 | Kd | 1 | 89257033 | L |
| TYISMLTLIF | 10 | Kd | 609 | 89257033 | |
| TYISMLTL | 8 | Kd | 9 | 89257033 | |
| TYVTNLNNL | 9 | Kd | 9 | 89257054 | |

| | | | | | |
|---|---|---|---|---|---|
| SYHFAFTTL | 9 | Kd | 4 | 89257066 | |
| WYLTLYTGL | 9 | Kd | 3 | 89257069 | |
| GYCTTEGEV | 9 | Kd | 10 | 89257087 | |
| CYHKNTSNNYL | 11 | Kd | 10 | 89257093 | |
| FYVTAIIYMFL | 11 | Kd | 23 | 89257093 | |
| IYICLLYL | 8 | Kd | 299 | 89257093 | M |
| IYICLLYLFDL | 11 | Kd | 638 | 89257093 | |
| IYLTNSAL | 8 | Kd | 12 | 89257093 | |
| IYLTNSALL | 9 | Kd | 327 | 89257093 | |
| IYMFLVVI | 8 | Kd | 498 | 89257093 | |
| IYMFLVVIYI | 10 | Kd | 673 | 89257093 | |
| IYVYFSVI | 8 | Kd | 198 | 89257093 | |
| LFITLRFI | 8 | Kd | 443 | 89257093 | M |
| VYFSVIFLAI | 10 | Kd | 290 | 89257093 | L |
| QHVYIGLTYI | 10 | Kd | 885 | 89257107 | |
| TYIFINIL | 8 | Kd | 70 | 89257107 | |
| TYIFINILL | 9 | Kd | 282 | 89257107 | |
| TYIFINILLL | 10 | Kd | 894 | 89257107 | L |
| VYIGLTYI | 8 | Kd | 19 | 89257107 | L |
| VYIGLTYIFI | 10 | Kd | 400 | 89257107 | |
| IYLLLLYSLI | 10 | Kd | 74 | 89257127 | L |
| RYLLFFSI | 8 | Kd | 41 | 89257127 | |
| RYLLFFSII | 9 | Kd | 42 | 89257127 | |
| RYLLFFSIIYL | 11 | Kd | 162 | 89257127 | |
| SYSGTFTGI | 9 | Kd | 4 | 89257128 | |
| FYFQTCSI | 8 | Kd | 6 | 89257148 | |
| LYLNYSSI | 8 | Kd | 31 | 89257149 | |
| LYLNYSSII | 9 | Kd | 320 | 89257149 | L |
| TYFLLLFSSEI | 11 | Kd | 38 | 89257149 | |
| YFLLLFSSEI | 10 | Kd | 228 | 89257149 | |
| SYFSFFSL | 8 | Kd | 13 | 89257164 | |
| SYFSFFSLV | 9 | Kd | 42 | 89257164 | |
| SYFSFFSLVI | 10 | Kd | 145 | 89257164 | |
| SYFSFFSLVII | 11 | Kd | 27 | 89257164 | |
| SYWSYFSFF | 9 | Kd | 807 | 89257164 | H |
| SYWSYFSFFSL | 11 | Kd | 16 | 89257164 | |
| QYIGVIASVI | 10 | Kd | 6 | 89257164 | |
| AFIAGTII | 8 | Kd | 795 | 89257175 | |
| LYAFIAGTI | 9 | Kd | 45 | 89257175 | |
| LYAFIAGTII | 10 | Kd | 63 | 89257175 | L |
| SYTFMLYAFI | 10 | Kd | 87 | 89257175 | M |
| IYMSGNNTRI | 10 | Kd | 6 | 89257178 | |
| IYILMMFI | 8 | Kd | 34 | 89257191 | |
| IYILMMFII | 9 | Kd | 72 | 89257191 | |
| IYILMMFIIAI | 11 | Kd | 57 | 89257191 | |
| KYLQIIYI | 8 | Kd | 30 | 89257191 | |
| KYLQIIYIL | 9 | Kd | 155 | 89257191 | |

| | | | | |
|---|---|---|---|---|
| MFIIAITMI | 9 | Kd | 747 | 89257191 |
| KYIQLFTQL | 9 | Kd | 2 | 89257199 |

| BALB/c

**Approach**

Clu
Aff
Aff
Aff
Clu
Clu
Clu
Clu
Clu
Clu
Clu
Clu
Clu
Clu
Aff
Aff
Aff
Aff
Clu
Clu
Clu
Clu
Clu
Clu
Aff
Aff
Aff
Clu
Clu
Clu
Clu
Clu
Clu
Clu
Clu
Clu
Aff
Aff
Aff

Aff
Aff
Aff
Aff
Aff
Clu
Clu
Clu
Clu
Clu
Clu
Clu
Clu
Aff
Aff
Clu
Clu
Clu
Aff
Aff
Aff
Aff
Clu
Clu
Aff
Aff
Clu
Clu
Clu
Clu
Clu
Clu
Clu
Clu
Aff
Aff
Aff
Aff
Clu
Clu
Clu
Clu
Clu
Clu
Clu
Clu
Clu

Clu
Clu
Clu
Clu
Clu
Clu
Aff
Aff
Aff
Aff
Aff
Aff
Aff
Clu
Clu
Clu
Aff
Clu
Clu
Clu
Clu
Clu
Clu
Clu
Aff
Clu
Clu
Clu
Clu
Clu
Clu
Clu
Clu
Clu
Clu
Clu
Clu
Clu
Clu
Clu
Clu
Clu
Clu
Clu
Aff
Clu
Clu

Clu
Clu
Clu
Clu
Clu
Clu
Aff
Clu
Clu
Clu
Clu
Clu
Aff
Aff
Clu
Clu
Clu
Clu
Clu
Clu
Aff
Clu
Clu
Clu
Clu
Aff
Aff
Clu
Clu
Clu
Aff
Aff
Clu
Clu
Aff
Clu
Clu
Clu
Clu
Clu
Aff
Aff
Clu
Clu
Clu
Clu
Clu

Clu
Clu
Aff
Clu
Clu
Clu
Clu
Clu
Clu
Clu
Aff
Clu
Aff
Clu
Clu
Clu
Aff
Clu
Clu
Clu
Aff
Clu
Clu
Clu
Clu
Aff
Clu
Clu
Clu
Clu
Clu
Aff
Clu
Clu
Clu
Aff
Aff
Clu
Clu
Clu
Aff
Aff
Aff
Aff
Aff
Clu
Aff

Aff
Aff
Clu
Clu
Clu
Clu
Clu
Aff
Aff
Aff
Aff
Clu
Aff
Aff
Aff
Aff
Aff
Aff
Clu
Clu
Clu
Clu
Clu
Clu
Clu
Aff
Aff
Aff
Clu
Clu
Clu
Aff
Aff
Clu
Clu
Clu
Clu
Clu
Clu
Aff
Aff
Aff
Aff
Aff
Clu
Clu
Clu

Clu
Aff
Aff
Clu
Clu
Clu
Aff
Clu
Clu
Clu
Clu
Clu
Clu
Clu
Clu
Clu
Clu
Clu
Clu
Aff
Aff
Clu
Clu
Clu
Clu
Clu
Clu
Aff
Clu
Clu
Clu
Clu
Clu
Clu
Aff
Aff
Clu
Clu
Aff
Clu
Clu
Aff
Clu
Clu
Clu
Aff
Aff

Aff
Aff
Aff
Clu
Clu
Clu
Clu
Clu
Clu
Clu
Clu
Clu
Clu
Clu
Clu
Clu
Clu
Clu
Clu
Clu
Clu
Clu
Clu
Clu
Aff
Aff
Clu
Clu
Clu
Clu
Clu
Clu
Clu
Clu
Clu
Clu
Aff
Clu
Clu
Clu
Clu
Aff
Clu
Clu
Clu
Clu
Clu

Clu
Aff
