# Supplementary material for: Clusters versus Affinity-Based Approaches in F. tularensis Whole Genome Search of CTL Epitopes
Source: PLoS One. 2012 May 1;7(5):e36440. doi: 10.1371/journal.pone.0036440 (PMC3341354; doi:10.1371/journal.pone.0036440)
Supplement: Table S5 — The list of peptides provides the compiled data of responders from previously identified responders (Subsets I and II; [14]) and every responder listed in Tables S1, S2, S3, S4. (PDF) [file pone.0036440.s005.pdf]

Table S5: Compilation of identified CTL epitopes

Sequence	Length	Predicted allele	Affinity (IC_{50}, nM)	Protein gi#	Subset/screen
KAYKNSYFL	9	Db	4	89255452	Subset V
LYLFAILPI	9	Kd	299	89255471	BALB/c
SYIINYKLLNI	11	Kd	421	89255563	Subset I
YKLLNINL	8	Db	669	89255563	Subset I
YKLLNINLI	9	Db	187	89255563	Subset I
LSPNQPILF	9	Dd	472	89255563	Subset IV
SAILNSKADI	10	Db	61	89255563	Subset IV
SYIINYKLLNI	11	Kd	421	89255563	BALB/c
IFLLMMYLM	9	Kb	627	89255570	Subset I
KYLGVPIFL	9	Kd	24	89255570	BALB/c
SSYQNVSYM	9	Db	6	89255591	Subset I
LTPYQAIAL	9	Kb	53	89255591	Subset IV
SYQNVSYM	8	Kd	116	89255591	BALB/c
ALMNWVIPL	9	Kb	334	89255626	Subset III
VLYYLVNLLTL	11	Kb	30	89255629	Subset III
YGYPYAYL	8	Kb	3	89255698	Subset V
VVFFAVKTAL	10	Kb	78	89255708	Subset II
RVLNLLFYL	9	Kb	552	89255715	Subset I
YSLLTAYIFM	10	Db	6	89255715	Subset I
YSLLTAYIF	9	Db	728	89255715	Subset I
LTAYIFMGGQL	11	Kb	729	89255715	Subset I
YQYVNPYIL	9	Db	7	89255720	Subset V
LIFSFVIM	8	Kb	140	89255729	Subset I
VIMVMLTGL	9	Kb	464	89255729	Subset I
GLLYIFKIGL	10	Kb	988	89255729	Subset I
VLFLNLYTIL	10	Kb	122	89255776	Subset I
LYTILLSFV	9	Kd	250	89255776	BALB/c
VWSIIYISL	9	Kb	329	89255790	Subset III
WSIIYISL	8	Kb	21	89255790	Subset III
SIIYISLGYL	10	Kb	482	89255790	Subset III
KYLKDTTESKI	11	Kd	10	89255800	BALB/c
FYIIITGI	8	Kd	7	89255814	BALB/c
KYCLLNEPI	9	Kd	6	89255818	BALB/c
IGFYSILL	8	Kb	599	89255853	Subset I
FYSILLTRIL	10	Kd	726	89255853	Subset I
FYSILLTRI	9	Kd	14	89255853	BALB/c
TNYFNYILPPL	11	Kb	8	89255886	Subset V
KNYQNLVM	8	Db	330	89255900	Subset III
ISYAKFAANTL	11	Kb	7	89255905	Subset V
SYINGTDGI	9	Kd	10	89255940	BALB/c
VLYFMGACI	9	Kb	865	89255979	Subset III

LYFMGACI	8	Kd	57	89255979	Subset III
LTYNYGYA	8	Kb	120	89255979	Subset III
IGYIKYLYYYG	11	Kb	398	89255996	Subset I
IFSMFFYIL	9	Kb	821	89255996	Subset I
ILIIFLYFEM	10	Kb	297	89255996	Subset I
IIFLYFEM	8	Kb	78	89255996	Subset I
LAFLFFFFFL	10	Kb	27	89255996	Subset I
IYLIIVQIILL	11	Kb	372	89255996	Subset I
KYLYYYGII	9	Kd	303	89255996	BALB/c
ICYVSTNIM	9	Kb	176	89256001	Subset I
CYVSTNIM	8	Kd	146	89256001	Subset I
TVFQTWLRL	9	Kb	118	89256001	Subset II
KVLVFFSSM	9	Kb	127	89256021	Subset III
VLVFFSSMLSL	11	Kb	313	89256021	Subset III
VFFSSMLSL	9	Kb	448	89256021	Subset III
VSLKYYFVLL	10	Kb	14	89256054	Subset I
LKYYFVLL	8	Kb	239	89256054	Subset I
YYVMILAVAI	10	Kd	25	89256057	BALB/c
QSALHFTLYL	10	Kb	668	89256070	Subset I
LIHYNLIFISL	11	Kb	80	89256070	Subset I
IHYNLIFISL	10	Kb	21	89256070	Subset I
IHYNLIFI	8	Kb	493	89256070	Subset I
RSLLKYFFVLL	11	Kb	79	89256087	Subset I
SLLKYFFVL	9	Kb	218	89256087	Subset I
FYFSIISYYI	10	Kd	23	89256094	BALB/c
LEFFYAYIAFL	11	Kb	216	89256124	Subset I
FYAYIAFLI	9	Kd	855	89256124	Subset I
AYIAFLIL	8	Kd	847	89256124	Subset I
AFLILLCYPM	10	Kb	885	89256124	Subset I
FLILLCYPM	9	Db	148	89256124	Subset I
LCYPMNIAAL	10	Kb	78	89256124	Subset I
FYNIIFSFI	9	Kd	7	89256124	BALB/c
IIILQYLLSPI	11	Kb	405	89256142	Subset I
LLYSLTTLL	9	Kb	141	89256177	Subset III
SILLYSLTTL	10	Kb	109	89256177	Subset III
SILLYSLTTLL	11	Kb	217	89256177	Subset III
ILLYSLTTLL	10	Kb	221	89256177	Subset III
ILLYSLTTL	9	Kb	110	89256177	Subset III
KGYNYGNYTLL	11	Kb	63	89256197	Subset III
SILIFAYL	8	Kb	10	89256199	Subset V
ISTVLYFIL	9	Kb	561	89256223	Subset I
LYFILTCYIAL	11	Kb	89	89256223	Subset I
ILTCYIAL	8	Kb	249	89256223	Subset I
ISMTVYYLVL	10	Db	162	89256227	Subset I
ISMTVYYL	8	Kb	32	89256227	Subset I
LNMHIKNFIMM	11	Kb	568	89256227	Subset I
ISFYGVLL	8	Kb	391	89256270	Subset I

FYGVLLIFFI	10	Kd	295	89256270	Subset I
ILFYLLVNL	9	Kb	63	89256270	Subset I
LLVNLNVFSSL	11	Kb	721	89256270	Subset I
LVNLNVFSSL	10	Kb	675	89256270	Subset I
FSSLNLVILPL	11	Db	96	89256270	Subset I
FYGVLLIFFI	10	Kd	295	89256270	BALB/c
LLAYLVAPSL	10	Kb	305	89256276	Subset III
ISINLGSLL	9	Kb	308	89256276	Subset III
SLLAYLVAPSL	11	Kb	94	89256276	Subset III
LAYLVAPSL	9	Kb	13	89256276	Subset III
FYLLTKDNI	9	Kd	4	89256308	BALB/c
LFIALQYPL	9	Kd	592	89256312	Subset I
LSMIAMTLLTM	11	Kb	336	89256315	Subset I
IAMTLLTM	8	Db	93	89256315	Subset I
TMFLQYIGI	9	Kb	75	89256315	Subset I
ISIIAAIFSYL	11	Kb	76	89256352	Subset I
SIIAAIFSYL	10	Kb	283	89256352	Subset I
SYLAALIAIII	11	Kd	81	89256352	BALB/c
ISIFLYLFL	9	Kb	45	89256377	Subset I
SIFLYLFL	8	Kb	54	89256377	Subset I
IIMLSLTYL	9	Db	105	89256404	Subset I
LSLTYLNTYKL	11	Kb	124	89256404	Subset I
KYIRGYFSL	9	Kd	4	89256410	BALB/c
SFFNYFKYMGM	11	Kb	103	89256429	Subset III
SFFNYFKYM	9	Kb	101	89256429	Subset III
FNYFKYMGM	9	Kb	42	89256429	Subset III
FKYMGMTLLAL	11	Kb	238	89256429	Subset III
TITQYFILL	9	Kb	134	89256432	Subset III
FILLNQYYQF	10	Db	328	89256432	Subset III
FILLNQYYQFL	11	Db	13	89256432	Subset III
ILLNQYYQFL	10	Kb	115	89256432	Subset III
LNQYYQFL	8	Kb	391	89256432	Subset III
YYQFLIQL	8	Kd	232	89256432	Subset III
NSYIINYKL	9	Kb	667	89256494	Subset III
ISFLYFMPII	10	Kb	55	89256517	Subset I
SFLYFMPIISL	11	Kb	59	89256517	Subset I
SFLYFMPII	9	Kb	692	89256517	Subset I
FLYFMPIISL	10	Kb	60	89256517	Subset I
LYFMPIISL	9	Kb	81	89256517	Subset I
SLFLGWIFL	9	Kb	615	89256517	Subset I
PSVQAYYWLL	10	Kb	728	89256552	Subset I
PSVQAYYWL	9	Kb	749	89256552	Subset I
SVQAYYWLL	9	Kb	843	89256552	Subset I
QIYSLMYLMM	10	Kb	240	89256552	Subset I
QIYSLMYLM	9	Kb	530	89256552	Subset I
LMYLMMFFAAL	11	Kb	11	89256552	Subset I
TILYFIAL	8	Kb	191	89256614	Subset I

TSYEFELL	8	Kb	5	89256631	Subset V
IALYTLISL	9	Kb	10	89256644	Subset V
LSAYANIMYYL	11	Kb	403	89256710	Subset I
FMLMNMGVVYL	11	Db	6	89256710	Subset I
MIILSVPL	8	Kb	367	89256710	Subset I
IAILFSFF	8	Kb	687	89256785	Subset I
AILFSFFSLSL	11	Kb	96	89256785	Subset I
ILFSFFSLSL	10	Kb	39	89256785	Subset I
ILFSFFSL	8	Kb	84	89256785	Subset I
SFFSLSLALL	10	Kb	619	89256785	Subset I
SLALLTFIAL	10	Kb	658	89256785	Subset I
AIMAMFESI	9	Kb	419	89256785	Subset III
QYILLCVI	8	Kd	12	89256788	Subset I
QYILLCVI	8	Kd	12	89256788	BALB/c
AYSPVMTII	9	Kd	7	89256799	BALB/c
IFISVVLL	8	Kb	479	89256811	Subset I
SFFYIFISVVL	11	Kb	167	89256811	Subset I
FYIFISVV	8	Kd	56	89256811	Subset I
YIFISVVLL	9	Kb	818	89256811	Subset I
IAIRYGNL	8	Kb	4	89256845	Subset V
IPILFSYSM	9	Kb	175	89256851	Subset I
SSYISVRIIM	10	Kb	64	89256892	Subset I
SYISVRIIM	9	Kd	39	89256892	Subset I
RIIMISITSL	10	Kb	800	89256892	Subset I
IIMISITSL	9	Kb	115	89256892	Subset I
ISITSLLITL	10	Db	55	89256892	Subset I
SITSLLITL	9	Kb	563	89256892	Subset I
SYISVRIIM	9	Kd	39	89256892	BALB/c
SIMSYYLL	8	Kb	16	89256893	Subset III
VLLSIMSYYLL	11	Kb	429	89256893	Subset III
LSIMSYYLL	9	Db	61	89256893	Subset III
SIMSYYLLL	9	Kb	124	89256893	Subset III
SSTTGYITIIL	11	Kb	886	89256896	Subset I
TTGYITIIL	9	Kb	748	89256896	Subset I
ILNLLYAQL	9	Kb	642	89256896	Subset I
LNLLYAQLFNL	11	Kb	309	89256896	Subset I
LNLLYAQL	8	Kb	5	89256896	Subset I
LYAQLFNLSSL	11	Kd	10	89256896	Subset I
GYISIPLAFI	10	Kd	32	89256896	BALB/c
GYITIILNLL	10	Kd	31	89256896	BALB/c
LYAQLFNL	8	Kd	360	89256896	BALB/c
IILSLALVLL	10	Kb	249	89256917	Subset I
LYMGLQYAFM	10	Kd	631	89256917	BALB/c
AIILYVPM	8	Kb	31	89256946	Subset I
IILYVPMSLSM	11	Kb	108	89256946	Subset I
SMFTVLYFLL	10	Kb	191	89256946	Subset I
YTITSYQNAL	10	Db	270	89256946	Subset III

VIIFLLVCL	9	Kb	167	89256946	Subset III
FSATNYYEVA	10	Db	294	89256946	Subset IV
SYQNALDMVII	11	Kd	99	89256946	BALB/c
VSIVNVTL	8	Db	98	89256968	Subset III
VNVTLVPLL	9	Kb	620	89256968	Subset III
IFLMFLSL	8	Kb	398	89256977	Subset I
LMFLSLTMLSP	11	Kb	700	89256977	Subset I
LMFLSLTM	8	Db	748	89256977	Subset I
LSLTMLSPL	9	Kb	58	89256977	Subset I
LSFTYVLASL	10	Kb	35	89256999	Subset III
VAIFILTYISM	11	Kb	21	89257033	Subset I
IFILTYISM	9	Kb	65	89257033	Subset I
TYISMLTLI	9	Kd	1	89257033	BALB/c
LIYVYFSV	8	Kb	14	89257093	Subset I
YVYFSVIFL	9	Db	107	89257093	Subset I
ILLAQTAKFL	10	Kb	829	89257093	Subset I
TVFYVTAIIYM	11	Kb	64	89257093	Subset I
VFYVTAIIYM	10	Kb	265	89257093	Subset I
IYICLLYLFDL	11	Kd	638	89257093	Subset I
IYICLLYL	8	Kd	299	89257093	Subset I
AALALAIIQL	10	Kb	126	89257093	Subset I
IYICLLYL	8	Kd	299	89257093	BALB/c
LFITLRFI	8	Kd	443	89257093	BALB/c
VYFSVIFLAI	10	Kd	290	89257093	BALB/c
VVLRQHVYIGL	11	Kb	86	89257107	Subset I
TYIFINILLL	10	Kd	894	89257107	BALB/c
VYIGLTYI	8	Kd	19	89257107	BALB/c
IYLLLLYSLI	10	Kd	74	89257127	BALB/c
LYLNYSSII	9	Kd	320	89257149	BALB/c
ISYWSYFSFF	10	Kb	462	89257164	Subset I
ISYWSYFSF	9	Kb	145	89257164	Subset I
SYWSYFSFFSL	11	Kd	16	89257164	Subset I
SYWSYFSFF	9	Kd	807	89257164	Subset I
WSYFSFFSL	9	Kb	24	89257164	Subset I
SYWSYFSFF	9	Kd	807	89257164	BALB/c
LYAFIAGTII	10	Kd	63	89257175	BALB/c
SYTFMLYAFI	10	Kd	87	89257175	BALB/c
QKYLQIIYIL	10	Kb	316	89257191	Subset I
KYLQIIYILM	10	Kb	788	89257191	Subset I
LQIIYILM	8	Kb	419	89257191	Subset I
